# Loss of Monoallelic Expression of *IGF2* in the Adult Liver *Via* Alternative Promoter Usage and Chromatin Reorganization

**DOI:** 10.3389/fgene.2022.920641

**Published:** 2022-07-22

**Authors:** Jinsoo Ahn, Joonbum Lee, Dong-Hwan Kim, In-Sul Hwang, Mi-Ryung Park, In-Cheol Cho, Seongsoo Hwang, Kichoon Lee

**Affiliations:** ^1^ Functional Genomics Laboratory, Department of Animal Sciences, The Ohio State University, Columbus, OH, United States; ^2^ The Ohio State University Interdisciplinary Human Nutrition Program, The Ohio State University, Columbus, OH, United States; ^3^ Animal Biotechnology Division, National Institute of Animal Science, Rural Development Administration, Jeonbuk, South Korea; ^4^ Columbia Center for Translational Immunology, Columbia University Irving Medical Center, Columbia University, New York, NY, United States; ^5^ National Institute of Animal Science, Rural Development Administration, Jeju, South Korea

**Keywords:** imprinting, IGF2, pigs, alternative promoter usage, biallelic conversion, chromatin reorganization

## Abstract

In mammals, genomic imprinting operates *via* gene silencing mechanisms. Although conservation of the imprinting mechanism at the *H19*/*IGF2* locus has been generally described in pigs, tissue-specific imprinting at the transcript level, monoallelic-to-biallelic conversion, and spatio-temporal chromatin reorganization remain largely uninvestigated. Here, we delineate spatially regulated imprinting of *IGF2* transcripts, age-dependent hepatic mono- to biallelic conversion, and reorganization of topologically associating domains at the porcine *H19/IGF2* locus for better translation to human and animal research. Whole-genome bisulfite sequencing (WGBS) and RNA sequencing (RNA-seq) of normal and parthenogenetic porcine embryos revealed the paternally hypermethylated *H19* differentially methylated region and paternal expression of *IGF2*. Using a polymorphism-based approach and omics datasets from chromatin immunoprecipitation sequencing (ChIP–seq), whole-genome sequencing (WGS), RNA-seq, and Hi-C, regulation of *IGF2* during development was analyzed. Regulatory elements in the liver were distinguished from those in the muscle where the porcine *IGF2* transcript was monoallelically expressed. The *IGF2* transcript from the liver was biallelically expressed at later developmental stages in both pigs and humans. Chromatin interaction was less frequent in the adult liver compared to the fetal liver and skeletal muscle. The duration of genomic imprinting effects within the *H19*/*IGF2* locus might be reduced in the liver with biallelic conversion through alternative promoter usage and chromatin remodeling. Our integrative omics analyses of genome, epigenome, and transcriptome provided a comprehensive view of imprinting status at the *H19*/*IGF2* cluster.

## 1 Introduction

Genomic imprinting operates in mammals as an epigenetic mechanism that leads to parent-of-origin-specific monoallelic expression of a subset of genes, mostly in a cluster, *via* silencing of either parental allele (Reik and Walter, 2001; Ferguson-Smith, 2011; Barlow and Bartolomei, 2014). Paternal or maternal expression of imprinted alleles is essential for embryonic development, animal growth and behavior, and diseases related to abnormal loss of imprinting (LOI) (Peters, 2014; Tian, 2014; Tucci et al., 2019). The allele-specific silencing is either direct (e.g., DNA hypermethylation on promoters (Ahn et al., 2021a) or indirect (e.g., by antisense non-coding RNAs (Latos et al., 2012) and chromatin insulators (Bell and Felsenfeld, 2000; Hark et al., 2000) and become complex when multiple types of silencing simultaneously occur on transcript isoforms generated by alternative promoter usage (Hayward et al., 1998a; Hayward et al., 1998b; Peters et al., 1999; Plagge et al., 2004; Ahn et al., 2020c). Regarding the insulators, studies have extensively investigated the *H19/Igf2* locus and established the insulator-mediated organization that coordinately regulates them *via* the imprinting control region (ICR) (Bartolomei et al., 1991; Dechiara et al., 1991; Ferguson-Smith et al., 1991; Bartolomei et al., 1993; Thorvaldsen et al., 1998; Bell and Felsenfeld, 2000; Hark et al., 2000). This ICR upstream (5′) of *H19* is methylated only in the paternal allele (i.e., paternally imprinted), so that the insulator CCCTC-binding factor (CTCF) is prevented from binding to the paternal allele of ICR and subsequently, the enhancer downstream (3′) of *H19* communicates with the far upstream promoter of *Igf2* to drive paternal *Igf2* expression. Insulin-like growth factor 2 (*IGF2*) is a growth factor that plays a central role in fetal and postnatal growth. Transgenic overexpression of the paternally expressed *Igf2* gene increased fetal growth (Sun et al., 1997), and upregulation or downregulation of *IGF2 via* aberrant imprinting is associated with the overgrowth disorder Beckwith-Wiedemann syndrome and the growth retardation disorder Silver-Russell syndrome, respectively (Jacob et al., 2013). The maternally expressed *H19* gene is a negative regulator of growth and encodes a tumor suppressor (Hao et al., 1993; Yoshimizu et al., 2008). This counteraction between paternally and maternally expressed genes posited in the parental conflict theory regulates balanced growth (Moore and Haig, 1991; Haig, 2004). Although the insulator model for the *H19/IGF2* locus has been established in mice and humans (Barlow and Bartolomei, 2014; Nordin et al., 2014), tissue- and transcript-specific imprinting and changes in chromatin organization during development remain to be identified in detail. These identifications can be achieved in a comparative manner in mammals that serve as biomedical models and are agriculturally important livestock. Pigs are relevant models for translational research due to their anatomical, physiological, as well as genomic similarities with humans (Lunney et al., 2021). Although studies have described DNA methylation and gene expression regarding *IGF2* and *H19* in pigs and a general conservation in the imprinting mechanism (Li et al., 2008; Park et al., 2009; Braunschweig et al., 2011; Criado-Mesas et al., 2019), tissue-specific imprinting at the transcript level, age-dependent hepatic monoallelic-to-biallelic conversion (loss of monoallelic expression), and spatio-temporal chromatin reorganization remain largely uninvestigated.

The multi-layered epigenetic regulatory machineries responsible for DNA methylation, chromatin accessibility, histone modifications, and gene expression can be investigated using integrative omics approaches. As the gold standard for DNA methylation analysis, whole-genome bisulfite sequencing (WGBS) of animal models including parthenogenetic embryos has been utilized to identify differentially methylated regions (DMRs) (Clark et al., 2006; Ahn et al., 2020a; Ahn et al., 2020c; Ahn et al., 2021b; a;Morrison et al., 2021). To assess chromatin accessibility (Thurman et al., 2012) and capture open chromatin sites, Assay for Transposase-Accessible Chromatin using sequencing (ATAC-seq) has been used (Buenrostro et al., 2013). Transcriptionally active promoters are marked by histone H3 trimethylated at lysine 4 (H3K4me3) (Howe et al., 2017). A subset of genes has an extended H3K4me3 signal, which covers the gene body and overlaps with the active enhancer mark—histone H3 acetylated at lysine 27 (H3K27ac), consisting of the broad epigenetic domain (Beacon et al., 2021). Cell type and tissue-specific enhancers in non-coding regulatory regions serve as key cis-regulatory elements for gene expression (Creyghton et al., 2010; Rada-Iglesias et al., 2011; Andersson et al., 2014; Coppola et al., 2016). During development, epigenetic modifications alter enhancer activities, as shown in H3K27ac enrichment followed by up-regulation of extracellular matrix genes which might reduce myogenic potential in aged skeletal muscle (Zhou et al., 2019). The formation of open chromatin regions and maintenance of enhancer elements are related to activation of tissue-specific genes (Xu et al., 2007; Wiench et al., 2011). Collectively, epigenetic modulations change chromatin structure and thereby alter DNA accessibility, which affects availability of enhancers and promoters to the transcriptional machinery. These epigenetic modifications might affect tissue-specific monoallelic gene expression within the *H19/IGF2* locus, which can be identified by profiling informative single nucleotide polymorphisms (SNPs) in genomic DNA and mRNA of the same individual (Ahn et al., 2021b; a). In addition, chromosomal conformation capture-based methods such as Hi-C have enabled unbiased identification of chromatin interactions across the genome (Lieberman-Aiden et al., 2009). The genome is partitioned into functional domains of different scale including megabase-long and evolutionarily conserved topologically associating domains (TADs) in which intra-domain chromatin interactions are frequent and cis-regulatory elements are coordinately regulated (Shen et al., 2012). The insulation score for genomic intervals along the chromosome is used to detect minima/valleys of insulation profile for areas of reduced chromatin interactions which are classified as TAD boundaries (Crane et al., 2015). As such, investigating the chromatin structure of imprinted domains in terms of TAD organizations (Lleres et al., 2019; Li et al., 2020) improves our understanding on imprinting clusters in the chromosomal context.

Here we aimed to delineate tissue-specific imprinting of major *IGF2* transcripts and hepatic mono- to biallelic conversion during development of pigs in comparison with humans. We found that the monoallelic-to-biallelic switch through liver-specific alternative promoter usage might occur concomitantly with removal of TAD boundaries and lower chromatin interaction frequencies at the porcine *H19/IGF2* locus. Our findings provided a comprehensive view of coordinated action of regulatory elements and chromatin organization and better understanding of tissue-specific and developmentally regulated genomic imprinting at the *H19/IGF2* locus.

## 2 Materials and Methods

### 2.1 Ethics Statement

Our experimental protocols for parthenogenetic studies in the pig were approved by the Institutional Animal Care and Use Committee of the National Institute of Animal Science, Rural Development Administration (RDA) of Korea (approval number NIAS 2015-670). Access to the National Bioscience Database Center (NBDC) Human Database for de-identified data was controlled to observe the Ohio State Human Research Protection Program (HRPP) policies on human subjects (study number 2020E1322).

### 2.2 Collection of Parthenogenetic and Control Embryos

Procedures of production of parthenogenetic embryos following *in vitro* maturation (IVM) of pig oocytes have been described in our previous reports (Kwon et al., 2017; Ahn et al., 2020b). In detail, ovaries of Landrace x Yorkshire x Duroc (LYD) pigs were obtained from a local slaughterhouse, transferred to our lab, and maintained in a thermos at 30–35°C. Cumulus-oocyte complexes (COCs) were gathered and washed in Tyrode’s lactate-Hepes medium containing 0.1% (w/v) polyvinyl alcohol. Before IVM, 50 COCs were washed three times in TCM-199 (GIBCO, Grand Island, NY, United States) [supplemented with 0.1% polyvinyl alcohol (w/v), 3.05 mM D-glucose, 0.91 mM sodium pyruvate, 0.57 mM cysteine, 0.5 μg/ml luteinizing hormone, 0.5 μg/ml follicle stimulating hormone, 10 ng/ml epidermal growth factor, 10% porcine follicular fluid (pFF), 75 μg/ml penicillin G, and 50 μg/ml streptomycin] and then placed in each well of five 4-well dishes (Nunc, Roskilde, Denmark) containing 500 µL of maturation medium and matured for 40–42 h at 38.5°C in an incubator containing 5% CO_2_. After maturation, cumulus cells were removed and oocytes having the first polar body were selected and activated as follows: oocytes were placed in a fusion chamber with 250 µm diameter wire electrodes (BLS, Budapest, Hungary) covered with 0.3 M mannitol solution containing 0.1 mM MgSO_4_, 1.0 mM CaCl_2_, and 0.5 mM Hepes and two DC pulses (1 s interval) of 1.2 kV/cm were applied for 30 µs using an LF101 Electro Cell Fusion Generator (Nepa Gene Co., Ltd. Chiba, Japan). Then, after 2 h of stabilization period, parthenogenetic embryos were placed into oviducts of two LY (Landrace X Yorkshire) surrogate gilts aged 12 months at onset of estrus to develop the embryos. Parthenogenetic embryos were recovered at day 21 from the surrogate gilts before they underwent morphological changes around day 30–35 (Bischoff et al., 2009; Hwang et al., 2020). As a control, fertilized embryos were also recovered at day 21 from gilts, after two LY gilts were naturally mated with boars twice with a 6 h interval during the natural heat period at the onset of estrus and confirmed pregnant by ultrasound examination. For the recovery, gilts were euthanized, and their reproductive tracts were sectioned, and the placenta was isolated from the uterus. Embryos were separated from the surrounding placenta and the surface of embryos was dried on cleaning tissues. Morphologically intact embryos with comparable sizes (approx. 2 cm) were selected and stored in liquid N2 until further use.

### 2.3 Whole-Genome Bisulfite Sequencing

Genomic DNA was isolated from whole collected embryos (triplicates for both parthenogenetic and control embryos) and fragmented. Accel-NGS Methyl-Seq DNA Library Kit (Swift Biosciences, Inc. Ann Arbor, MI, United States) was used to optimize bisulfite conversion of genomic DNA according to the manufacturer`s instructions. PCR was conducted to amplify the bisulfite-treated DNA with adapter primers, Diastar™ EF-Taq DNA polymerase (Solgent, Daejeon, Korea), and the following thermal cycles: 3 m at 95°C followed by 35 cycles of 30 s at 95°C, 30 s at 60°C, and 30 s at 72°C, and a final extension for 5 m at 72°C. After bead-based clean-up, libraries were sequenced on an HiSeqX sequencer by Macrogen (Seoul, Korea) with 151 bp paired-end reads. Data quality was checked using FastQC (v0.11.7). Raw reads in FASTQ format were quality- and adapter-trimmed with the default parameters of Trim Galore (v0.4.5), except for additional trimming of 18 bp off the 3′ end of R1 and the 5′ end of R2 for removing bases derived from the sequence tag introduced in the library preparation procedure (--three_prime_clip_R1 18 --clip_R2 18). Trimmed reads (more than 800 million reads for each sample) were aligned to the pig reference genome (Sscrofa11.1, GenBank accession: GCF_000003025.6) using Bismark (v0.22.3) with default parameters including --no_overlap for paired-end reads (Krueger and Andrews, 2011). After deduplication using the deduplicate_bismark command, the Bismark methylation extractor was used to calculate methylation percentage of every cytosine in CpG context. Next, the DMR caller, metilene (v0.2-8), was used to identify *de novo* DMRs with default parameters [including maximum distance of 300 bp between CpGs (-M 300), minimum of 10 CpGs (-m 10), and minimum mean methylation difference of 0.2 (−d 0.2)] and a false discovery rate (FDR) option (Juhling et al., 2016). Methylation ratios and DMRs (FDR < 0.05) were visualized on genomic coordinates using the R/Bioconductor package Gviz (v1.28.3) (Hahne and Ivanek, 2016).

### 2.4 RNA Sequencing

Total RNA from whole embryo samples (*n* = 3 for each of the control and parthenote) was isolated with TRIzol reagent (Sigma-Aldrich, United States) following the manufacturer’s instructions. The RNA samples were treated with DNase I to avoid genomic DNA contamination and electrophoresed in 1.2% agarose gels to evaluate the integrity of RNA, which was confirmed by 28S/18S rRNA ratio > 2 and RNA integrity number (RIN) > 7 using an Agilent 2100 BioAnalyzer. The concentrations of RNA were assessed by the ratios of A260/A280 and A260/A230 (1.8–2.0). One ug of total RNA and the TruSeq RNA Sample Prep Kit v.2 (Illumina, San Diego, CA, United States) were used to construct cDNA libraries, and final libraries were produced using the protocol consisting of polyA-selected RNA extraction, RNA fragmentation, random hexamer primed reverse transcription and amplification. The cDNA libraries were quantified by quantitative Real-Time PCR (qPCR), and qualification of the libraries was assessed using the Agilent 2100 Bioanalyzer. The library products (100 nt paired-end) were sequenced by the Illumina HiSeq2500 RNA-Seq platform. The raw RNA sequencing reads (FASTQ format) were checked for quality by FastQC (v0.11.7) and trimmed and filtered by Trimmomatic v0.38 with default parameters (Bolger et al., 2014). Then, using STAR aligner (v2.7.5) with default parameter settings (Dobin et al., 2013), cleaned sequencing reads were mapped to the pig reference genome sequence (Sscrofa11.1). Duplicated reads were removed using Picard MarkDuplicates and reads were filtered using SAMtools (-q 30) (Li et al., 2009). Read coverages in BAM files were normalized to values equivalent to transcripts per million (TPM) using bamCoverage in deepTools (v3.5.0) with parameters (--binSize 10, --smoothLength 15) (Ramirez et al., 2014) and plotted using the R/Bioconductor package Gviz (v1.28.3) (Hahne and Ivanek, 2016).

### 2.5 Analyses of Differential Gene Expression

Raw RNA-seq reads in the FASTQ format were quantified against indexed pig transcriptome using Salmon (v1.3.0) in the mapping-based mode (Patro et al., 2017). TPM values of each gene were obtained for parthenogenetic embryos (PA) and control embryos (CN) (*n* = 3 for each) from Salmon output files (quant.sf). The output files were then imported by tximport function to construct a gene-level DESeqDataSet object for the R/Bioconductor package DESeq2 (v1.28.1) (Love et al., 2014). The test for DEGs was conducted by DESeq2. To obtain significant DEGs, combined criteria of FDR < 0.05 and the absolute log2-fold change > 1 were used, where a fold change is defined as read counts in PA divided by read counts in CN.

### 2.6 Profiling Gene Regulatory Elements

Raw FASTQ files deposited with GEO accession number GSE158430 (ATAC-seq, and H3K27ac, H3K4me3, and CTCF ChIP-seq) for 6-month-old pigs (Kern et al., 2021), GSE143288 (ATAC-seq, and H3K27ac and H3K4me3 ChIP-seq) for 2-week-old pigs (Zhao et al., 2021), GSE153452 (CTCF-seq) for pig embryonic fibroblasts (Li et al., 2020), and GSE155324 (CTCF-seq) for human lymphoblasts (Ushiki et al., 2021) were downloaded *via* the European Nucleotide Archive (ENA) Globus GridFTP. The quality of the raw sequencing reads was checked using FastQC (v0.11.8), and raw reads were trimmed and filtered using Trimmomatic (v0.38) with default settings (Bolger et al., 2014). All trimmed reads were mapped to the pig reference genome (Sscrofa11.1) or the human reference genome (GRCh38.p13, RefSeq assembly accession: GCF_000001405.39) using BWA-MEM aligner (v 0.7.17-r1198) using default parameters (Li, 2013). For ATAC-seq, mitochondrial genome was removed from the pig genome before alignment to avoid contamination of the mitochondrial genome which is more accessible owing to chromatin packaging deficiencies (Yan et al., 2020). Aligned reads were deduplicated using Picard MarkDuplicates, and filtered for quality using SAMtools (MAPQ > 30) (Li et al., 2009). MACS2 was used with default parameters to call peaks except for broad peaks (--broad) for ATAC-seq and FDR < 0.01 (-q 0.01) (Zhang et al., 2008). Read coverages in BAM files were normalized to 1x depth (reads per genomic content, RPGC) using bamCoverage in deepTools (v3.5.0) with parameters (--binSize 10, --smoothLength 15) (Ramirez et al., 2014). Peaks were visualized on genomic coordinates using the R/Bioconductor package Gviz v1.36.2 (Hahne and Ivanek, 2016).

### 2.7 Analyses of Tissue-Specific and Developmental Stage-Specific Expression

Raw RNA-seq data of normal pigs [PRJEB44486 (under Sus scrofa section in the FAANG datasets, https://data.faang.org/dataset), GSE77776 (Li et al., 2017), PRJNA493166 (Zhang et al., 2019), GSE158430 (Kern et al., 2021), PRJNA597972, GSE124484, GSE92433, PRJNA721126, GSE93855 (Tang et al., 2017), and GSE157045 (Yang et al., 2021)] and humans [GSE63634 (Yan et al., 2016), SRP166862 (George et al., 2019), PRJNA395106, and GSE120795 (Suntsova et al., 2019)] were downloaded through ENA’s Globus GridFTP, except for raw data files of the human adult liver (accession hum0158.v2 for controlled access) which were downloaded *via* SFTP of the NBDC Human Database. The RNA-seq processing procedures were the same as above.

### 2.8 Analyses of Allele-Specific Expression

Datasets with genomic DNA and mRNA sequencing data from the same individuals were used. In detail, in addition to the above RNA-seq data, raw data for genomic DNA from the same individual were also downloaded from ENA’s Globus GridFTP, except for the human adult liver obtained from NBDC’s SFTP. For each pig breed, one liver and skeletal muscle sample from a 60-day-old pig was used (PRJNA309108/GSE77776) (Li et al., 2017). The human fetal liver samples were from two fetuses at 12 weeks after gestation (GSE63634) (Yan et al., 2016). The IDs of human liver samples from adults aged 31–74 were RK001, RK003, RK018, RK019, RK024, RK075, RK130, RK141, and RK157 (hum0158.v2). The IDs of human smooth muscle samples from reproductive age adults were MP100N, MP136N, MP169N, NW206N, and GO537N (SRP163897/SRP166862) (George et al., 2019). The IDs of human lung samples of adults aged 68–77 were N1, N3, N5, N12, N19, and N23 (PRJNA395106). Individuals with these IDs had heterozygous SNPs at the *IGF2* locus in genomic DNA. In addition, an RNA-seq dataset, PRJNA597972, was used to analyze biallelic tendencies.

Whole-genome sequencing (WGS), whole-exome sequencing (WES), and H3K4me1 ChIP-seq data were cleaned and aligned using BWA-MEM as above. The deduplicated BAM files were used to detect SNPs and obtain allele counts in individual samples by generating vcf files through bcftools mpileup piped to bcftools call command. The published pig SNP data (GCA_000003025.6_current _ids.vcf.gz) were obtained from the EBI ftp server (ftp://ftp.ebi.ac.uk/pub/databases/eva/rs_releases/release_2/by_species/sus_scrofa/Sscrofa11.1/). The human SNPs in the vcf file (GCF_000001405.39.gz) for the GRCh38.p13 genome were downloaded from the NCBI data repository (https://ftp.ncbi.nih.gov/snp/.redesign/latest_release/VCF/). For sequencing to generate chromatogram for *IGF2(8)* and *IGF2-AS*, genomic DNA and RNA were isolated from the liver of 6-month-old Berkshire pigs. To amplify a DNA fragment containing a potential SNP on the second exon of the *IGF2(8)* transcript, primers were designed on the first intron (forward: 5′-AGCGTGGA GAGGCTCTCTT-3′) and the second intron (reverse: 5′-ACC​CAA​ACA​CTC​AAT​GCA​GCT​TT-3′). For a potential SNP on the second exon of the *IGF2-AS* transcript, primers were designed on the first intron (forward: 5′-CTG​CTC​TGG​GTT​CCC​CAT-3′) and the second intron (reverse: 5′-CTGACAA CCCTGCCCTGTT-3′). After sequencing and confirming heterozygosity of the SNP, primers for cDNA were designed on the first exon (forward: 5′-CCC​CAT​TGG​CAC​CAG​TAC​AG-3′) and the third exon (reverse: 5′-GCT​GAG​CCC​GAG​GAG​ATG​TG-3′) of *IGF2(8)* and the first exon (forward: 5′-GGA​CAC​GCG​AGG​CGA-3′) and second exon (reverse: 5′-CAA​GGT​CCA​GGC​GCA​TGT-3′) of *IGF2-AS* to avoid genomic DNA contamination. PCR was conducted using the Taq DNA Polymerase (#M0273S, New England Biolabs, Ipswich, MA, United States) with an initial incubation at 95°C for 30 s, followed by 43 cycles at 95°C for 30 s, 56°C for 35 s, and 68°C for 20 s. The final extension was performed at 68°C for 5 min. After agarose gel electrophoresis, DNA was extracted from separated PCR bands using a QIAquick Gel Extraction Kit (#28104, Qiagen, Venlo, Netherlands) according to the manufacturer’s protocol and sent out for Sanger sequencing at The Ohio State University Core Facility.

### 2.9 Hi-C Data Processing

Raw Hi-C data in FASTQ format of the liver of fetal (embryonic day 90) and adult (2-years-old) Bamaxiang pigs (PRJNA482496) (Tian et al., 2020) and skeletal muscle tissues of a Luchuan pig (embryonic day 35) (GSE166346) (Yuan et al., 2021) and Large White pigs (2-week-old) (GSE143288) (Zhao et al., 2021) were retrieved through ENA’s Globus GridFTP. After assessing the quality of data using FastQC (v0.11.7), the raw paired-end reads were trimmed and filtered out to remove low quality reads, adapters, and reads shorter than 20 bp by using default settings of Trim Galore (v0.4.5). Cleaned data were processed using HiC-Pro (v.3.1.0) with default parameters (Servant et al., 2015) while specifying the index for bowtie2 (v2.4.4) and MboI (or DpnII) restriction fragments according to data submitters’ publications (Tian et al., 2020; Yuan et al., 2021; Zhao et al., 2021). To determine concordance of raw Hi-C matrices, GenomeDISCO was used to produce smoothed matrices and randomly work on the smoothed matrices to obtain concordance scores (Ursu et al., 2018). The validPairs files from matrices with high concordance were merged to increase resolution and normalized by iterative correction and eigenvector decomposition (ICE) using parameters of HiC-Pro (-s merge_persample -s build_contact_maps -s ice_norm). TADs were identified using insulation scores and ICE normalized matrices were visualized using the GENOVA R-package (Van Der Weide et al., 2021).

## 3 Results

### 3.1 A Differentially Methylated Region Within the Porcine *H19/IGF2* Locus is Paternally Methylated at a CpG Island

**FIGURE 1:**
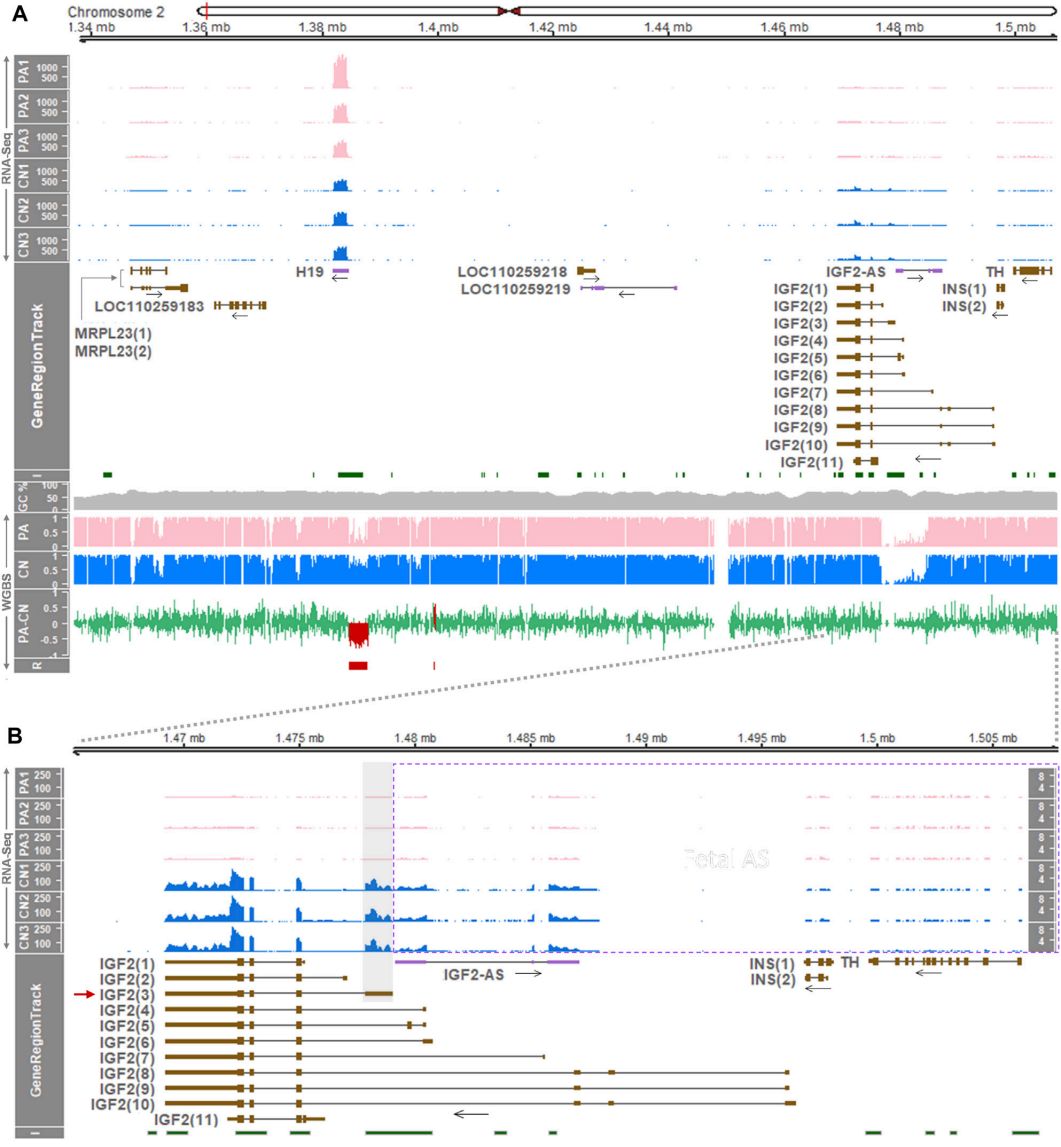
Gene expression and DNA methylation profiling at the *H19*/*IGF2* locus in the porcine embryo. **(A)** Above GeneRegionTrack, normalized read coverages from RNA-Seq are shown as transcripts per million (TPM; y-axis) in each track of PA and CN embryos. In GeneRegionTrack, genes located in the 0.171-Mb (171-Kb) region between the *MRPL23* and *TH* genes (chr2:1,337,000-1,508,000) are displayed by either brown boxes [tall, translated regions; short, untranslated regions (UTRs)] for protein-coding transcripts or purple boxes for non-coding transcripts. The directions of transcription are marked by horizontal arrows. Below GeneRegionTrack, averages of cytosine methylation ratios (*n* = 3, PA and CN each) obtained by WGBS are shown. PA-CN indicates mean methylation ratios of PA subtracted by those of CN. R represents DMRs called by the metilene software (FDR < 0.05) which are also overlaid in the PA-CN track. I (CpG islands) and GC% (GC content) were derived from the UCSC Table Browser. **(B)** The locus spanning *IGF2*, *IGF2-AS*, *INS*, and *TH* genes (43-Kb, chr2:1,465,000-1,508,000) were zoomed in. For *IGF2-AS*, *INS*, and *TH*, expression is displayed within a dotted rectangle and an additional y-axis (TPM) is shown at the far right. Pig gene transcripts include *MRPL23(1)*, XM_021083608.1; *MRPL23(2)*, XM_021083607.1; *IGF2(1)*, XM_021080593.1; *IGF2(2)*, XM_021080637.1; *IGF2(3)*, XM_021080576.1; *IGF2(4)*, XM_021080612.1; *IGF2(5)*, XM_021080582.1; *IGF2(6)*, XM_021080648.1; *IGF2(7)*, XM_021080641.1; *IGF2(8)*, XM_021080607.1; *IGF2(9)*, XM_021080643.1; *IGF2(10)*, XM_021080603.1; *IGF2(11)*, XM_021080596.1; *INS(1)*, XM_021081278.1; *INS(2)*, NM_001109772.1.

Using diploid uni-maternal PA embryos and bi-parental CN embryos, we performed WGBS at an approximately 50X depth (Supplementary Table S1). By analyzing the WGBS data, DMRs between the embryos were obtained whose mean methylation difference (i.e., a mean of PA subtracted by a mean of CN) was more than 0.2 (hypermethylation in PA) or less than 0.2 (hypomethylation in PA) with significance (FDR < 0.05) (Supplementary Figure S1A). Compared with methylated regions without significance, DMRs tended to be longer in base pairs and greater in CpG numbers, as a result of processing approximately 588 million deduplicated uniquely mapped reads for each replicate on average (Supplementary Figure S1B and Supplementary Table S1). Using unmethylated lambda phage DNA added to sample DNA prior to fragmentation, bisulfite conversion rates at CpG, CHG, and CHH sites were calculated and estimated to be 99.69%–99.71% across samples representing successful library construction (Supplementary Table S1). In order to examine DNA methylation status within the *H19*/*IGF2* locus in porcine embryos, methylation ratios at single-base resolution were analyzed. Based on the mean methylation ratio at each CpG site, diploid uni-maternal PA carried a broad range of hypomethylation immediately upstream (5ʹ) of the non-coding *H19* gene (Figure 1A bottom panel, Supplementary Table S2). Hemi-methylation occurred in bi-parental CN, and a DMR between PA and CN was identified which mostly overlapped a CpG island (Figure 1A bottom panel). It was consistent with a previous study regarding the presence of the porcine *H19*-DMR, where maternal alleles were almost unmethylated and paternal alleles were completely methylated which led to hemi-methylation (Braunschweig et al., 2011). Additionally, a narrow hypermethylated DMR in PA was located farther upstream of *H19* (Figure 1A bottom panel). DNA methylation in the upstream and downstream of the *IGF2*, *INS*, *TH* genes did not show differences between PA and CN, and various *IGF2* transcripts did not exhibit a transcript-specific methylation pattern (Figure 1A bottom panel). In summary, the hypomethylated region in PA (i.e., paternally methylated *H19*-DMR) was the only DMR that overlapped a CpG island throughout the *H19*/*IGF2* locus of the pig embryo, and transcript-specific DMRs were not found in the *IGF2* locus.

### 3.2 Expression of the *IGF2* Gene in Pig Embryos is Paternal Allele-Specific

In our model of PA and CN, gene and transcript expressions within the *H19*/*IGF2* locus were examined to investigate their imprinting status. Analyses of differentially expressed genes (DEGs) on RNA-seq data (Supplementary Table S1) revealed that expression of the non-coding *H19* gene tended to increase in PA compared to CN (1.61-fold higher in PA) suggesting its maternal expression, i.e., had a higher expression in two maternal alleles of PA than in one maternal allele of CN (Figure 1A top panel, Supplementary Table S3, and Supplementary Figure S1C). This deviation from a 2-fold increase might be accounted for by gene dosage compensation in diploid uni-parental embryos or loss of imprinting (Shemer et al., 1996; Park et al., 2011). Expression of the *IGF2* genes was almost exclusive in CN embryos (adjusted *p*-value < 0.001), indicating expression in the paternal allele of CN while being absent in PA without the paternal allele (Figure 1B; Supplementary Table S3, and Supplementary Figure S1C). Among the *IGF2* transcript isoforms, the major transcript was *IGF2(3)* (short-form; GenBank accession number: XM_021080576.1) having four exons and its non-overlapping first exon carried predominant read coverages (Figure 1B). In addition, paternal expression of antisense of *IGF2* (*IGF2-AS*) was indicated by almost exclusive read coverages in CN (adjusted *p*-value < 0.001) (Figure 1B; Supplementary Table S3, and Supplementary Figure S1C). Other genes and gene transcripts including *MRPL23* and *INS* appeared to be expressed biallelically and expression of the *TH* gene tended to increase in PA embryos, while expression of LOC110259183, LOC110259218, and LOC110259219 was almost undetectable (Figure 1; Supplementary Table S3, and Supplementary Figure S1C). Consequently, the imprinted expressions of the *IGF2(3)* and *IGF2-AS* genes were shown to be paternal monoallelic.

### 3.3 Regions Surrounding the Porcine *H19* and *IGF2* Locus Accumulate Distinguishable Gene Regulatory Elements Between the Liver and Skeletal Muscle

**FIGURE 2:**
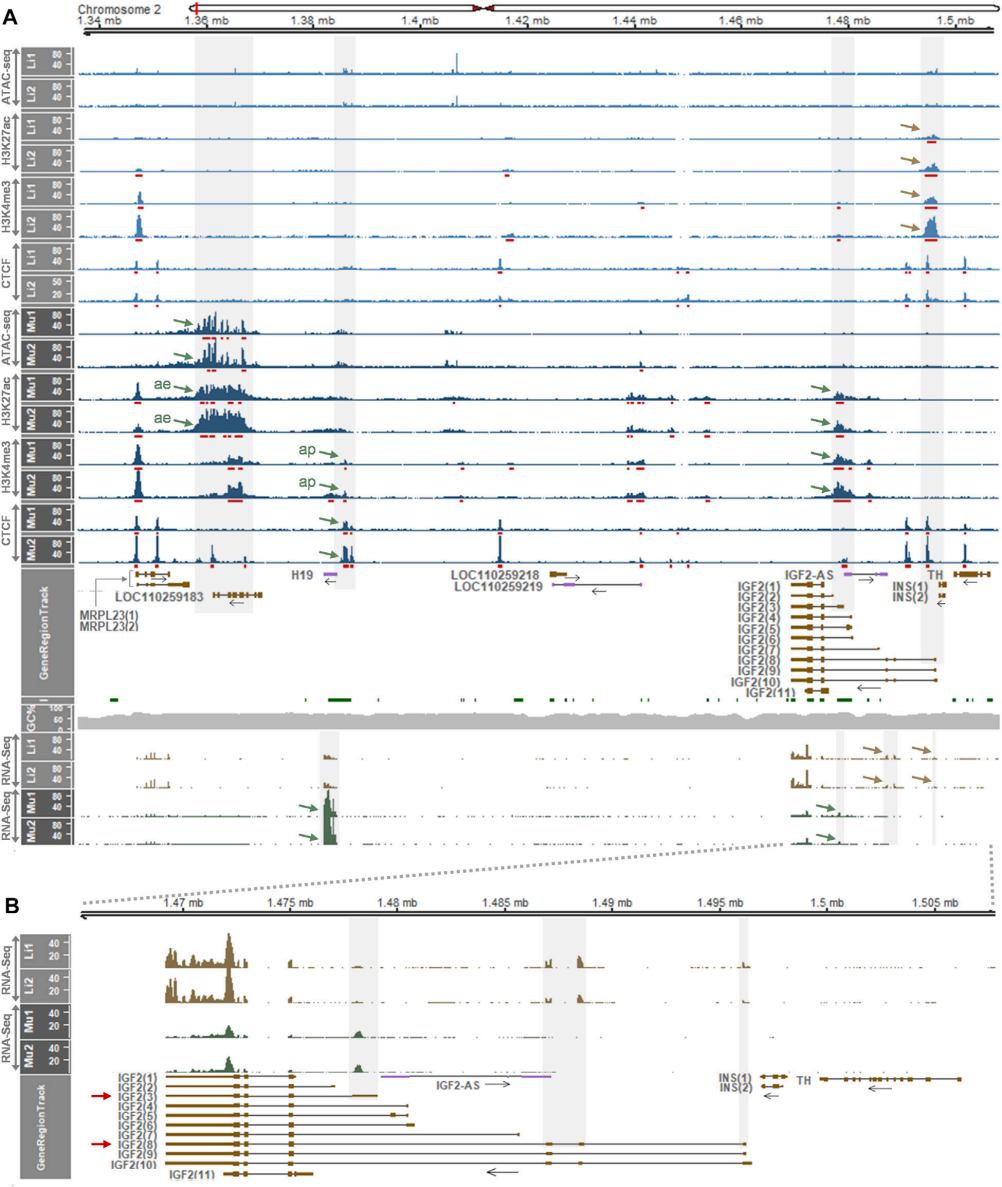
Epigenetic regulatory elements identified in the *H19/IGF2* locus of liver and skeletal muscle of 6-month-old pigs and gene expression profiling. **(A)** Above GeneRegionTrack, peaks of ATAC-seq, H3K27ac, H3K4me3 and CTCF are displayed in 1x normalized read coverages. MACS2-called peaks are underscored with red bars. Peaks of interest in the liver and skeletal muscle are pointed with brown and blue arrows, respectively. ae, active enhancer; ap, active promoter. Below GeneRegionTrack, normalized read coverages in TPM values from RNA-Seq from the same pigs (P348 and P350) are displayed. Li1, liver 1 from P348; Li2, liver 2 from P350; Mu1, skeletal muscle 1 from P348; Mu2, skeletal muscle 2 from P350. **(B)** RNA-Seq read coverages in the *IGF2* locus are zoomed in. Data were retrieved from a dataset (GSE158430). Details about y axis titles and the plot are in Figure 1 legend.

To examine whether imprinted monoallelic expression at the *IGF2* locus is maintained in different developmental stages, we first analyzed expression levels *H19* and *IGF2* in multiple tissues of 6-month-old pigs using a dataset retrieved by the Gene Expression Omnibus (GEO) accession GSE158430. At this stage, expression of *IGF2* was prominent in the liver and expression of *H19* was high in skeletal muscle (Supplementary Figure S2). In other pig tissues, including the adipose tissue, brain hypothalamus, lung, and spleen, expression of both *IGF2* and *H19* was substantially low (Supplementary Figure S2). Therefore, we investigated gene regulatory elements in the liver and skeletal muscle of the same pigs within and near the porcine *H19/IGF2* locus that might affect gene expression. ATAC-seq (for open chromatin), H3K27ac (at active enhancers and promoters), H3K4me3 (at active promoters), and CTCF (insulators) were analyzed. Our analysis of these datasets from two biological replicates of skeletal muscle revealed that H3K27ac signals were distributed around ATAC peaks spanning ∼20 kb (chr2:1,346,472-1,366,301) approximately 15–35 kb downstream (3′) of the *H19* transcription start site (TSS) (ae, Figure 2A), suggestive of active enhancers. However, these signals were absent in the liver. On the other hand, H3K27ac peaks upstream (5′) of *IGF2(8)* were enriched with H3K4me3 in the liver, and also H3K27ac peaks upstream of *IGF2(3)* were enriched with H3K4me3 in skeletal muscle (approx. 1–2 kb H3K27ac and H3Kme3 signals) (Figure 2A), suggesting that they represent regulatory features including active promoters. In addition, in skeletal muscle but not in the liver, H3K4me3 signals located immediate upstream (5′) of *H19* where CTCF peaks co-localized (ap, Figure 2A). It indicated that the *H19* promoter was activated, and this activation might be attributed to the aforementioned active enhancer downstream of *H19* and monoallelic CTCF binding which leads to monoallelic activation of the *H19* promoter as previously reported regarding the human and mice ICRs (Bell and Felsenfeld, 2000; Hark et al., 2000). Moreover, although monoallelic CTCF binding in the skeletal muscle was not confirmed due to lack of heterozygous sites, the monoallelic CTCF binding was evident in pig embryonic fibroblasts (PEFs) whose CTCF sites were analogous to those of human lymphoblasts (Supplementary Figure S3). Noticeably, expression of *H19* was low in the liver and high in skeletal muscle (Figure 2A bottom panel, and Supplementary Figure S2), and *IGF2* transcripts expressed in this locus were different between the two tissues: *IGF2(8)* in the liver and *IGF2(3)* in skeletal muscle, and total expression of *IGF2* was higher in the liver (Figure 2A bottom panel, Figure 2B, and Supplementary Figure S2).

**FIGURE 3:**
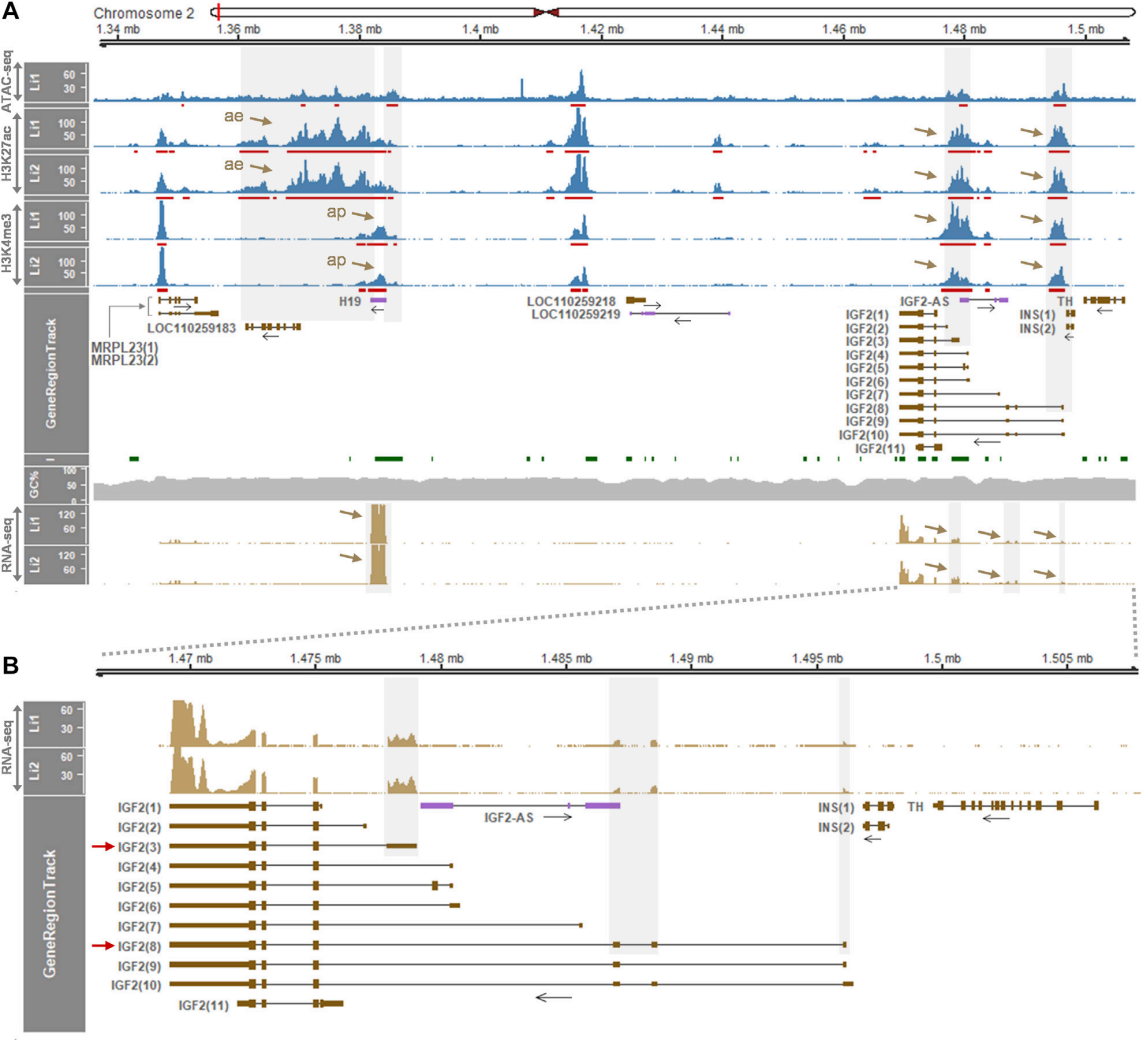
Enrichment of gene regulatory signals and expression of genes within the *H19/IGF2* locus of the liver of 2-week-old pigs. **(A)** ATAC-seq, H3K27ac, and H3K4me3 signals in the liver (Li) of two Large White pigs are represented with 1x normalized read coverages. Red bars indicate MACS2-called peaks. Both grey shades and brown arrows denote gene regulatory signals and gene expression. ae, active enhancer; ap, active promoter. **(B)** Expression pattens of the *IGF2* transcripts is zoomed in. Both *IGF2(3)* and *IGF2(8)* are expressed in the same liver tissues of the 2-week-old pigs. Data were retrieved from GSE143288.

We further investigated an additional presence of gene regulatory elements in the liver which deviate from the insulator model (Barlow and Bartolomei, 2014; Nordin et al., 2014) using another dataset (GSE143288). In 2-week-old pigs, H3K27ac peaks were distributed in the downstream of *H19* around the open chromatin region indicated by ATAC signals, although the dataset contained ATAC data for the one pig (Li1) (Figure 3A top panel). The H3K4me3 signals near *H19* indicated an activated promoter, and all the ATAC, H3K27ac, and H3K4me3 signals around the first exon of *IGF2(3)* suggested the long-range insulator-mediated regulation of *IGF2(3)* expression. However, those ATAC, H3K27ac, and H3K4me3 signals were also detected near the first exon of the *IGF2(8)* transcript (Figure 3A top panel), suggesting the presence of an additional transcript-level gene regulation. Consistently, the expression of both *IGF2(3)* and *IGF2(8)* was observed in the same liver tissues while *H19* was expressed in the far downstream of *IGF2* (Figure 3A bottom panel and Figure 3B). Taken together, it suggested that, compared to the *IGF2(3)* expression in the whole embryo (Figure 1) and the *IGF2(8)* expression in the liver from 6-month-old pigs (Figure 2), both *IGF2(3)* and *IGF2(8)* are expressed in the liver of two-week-old pigs (Figure 3) under the regulation of two different sets of gene regulatory elements.

### 3.4 Developmental Changes of the Porcine *IGF2* Gene Expression and Bi- or Mono-Allelic Expression are Distinct in the Liver and Skeletal Muscle

**FIGURE 4:**
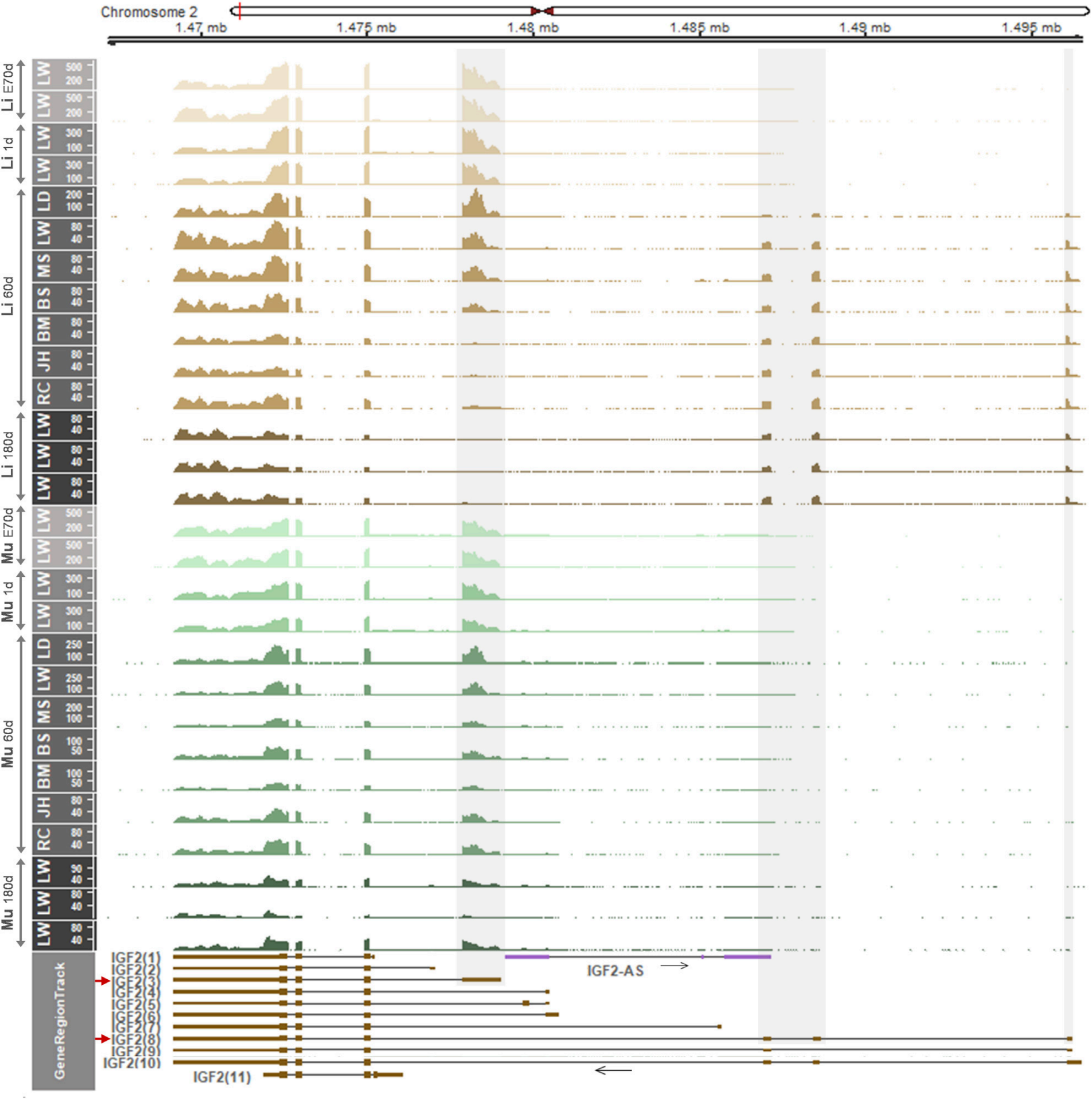
mRNA expression in the liver and skeletal muscle within the *IGF2* locus of embryonic day 70 (E70 d), 1-day-old (1 d), 60-day-old (60 d), and 180-day-old (180 d) pigs. Pig RNA-seq data generated using embryonic liver and skeletal muscle (PRJEB44486), 1 d liver and skeletal muscle (PRJEB44486), 60 d liver and skeletal muscle (GSE77776), and 180 d liver and skeletal muscle (PRJNA493166) were analyzed. Developmental stages are separated and indicated for liver and skeletal muscle using light brown to dark brown and light green to dark green, respectively. Non-overlapping exons with high read coverages are overlaid with grey shades and corresponding predominantly expressed transcripts (*IGF2(3)* and *IGF2(8)*) are marked by red arrows. Li, liver; Mu, skeletal muscle. LW, Large White; LD, Landrace; MS, Meishan; BS, Berkshire; BM, Bamei; JH, Jinhua; RC, Rongchang pigs.

Considering the aforementioned distinct gene regulation and expression patterns in the liver, we examined whether the *IGF2* gene expression is regulated developmentally and shows an allele-specific pattern. First, using RNA-seq datasets, read coverages for *IGF2* transcripts were analyzed in the liver and skeletal muscle across developmental stages. In both the liver and skeletal muscle, on embryonic day 70 and postnatal day 1, the *IGF2(3)* transcript was predominantly expressed (Figure 4). On day 60, however, the *IGF2(3)* transcript was not always predominant in the liver of analyzed pigs, while in the skeletal muscle the *IGF2(3)* transcript was predominant (Figure 4). In particular, in the liver of Landrace pigs, the *IGF2(3)* transcript was predominant and the *IGF2(8)* expression was detected at a low level. In the liver of Large White and Meishan pigs both *IGF2(3)* and *IGF2(8)* transcripts were predominant, while Berkshire pigs showed high expression of the *IGF2(8)* transcript and reduced expression of the *IGF2(3)* transcript. On the other hand, expression of the *IGF2(3)* transcript was almost absent in the liver of Bamei, Jinhua, and Rongchang pigs, whereas the *IGF2(8)* transcript was predominant (Figure 4). On day 180, the *IGF2(8)* transcript was predominant in the liver and the *IGF2(3)* transcript was predominant in skeletal muscle (Figure 4). The total read coverages of *IGF2* tended to decrease in both the liver and skeletal muscle during development. The transition of *IGF2* expression in the liver, but not in skeletal muscle, was repeatedly shown in additional data (Supplementary Figures S4A and S5).

**FIGURE 5:**
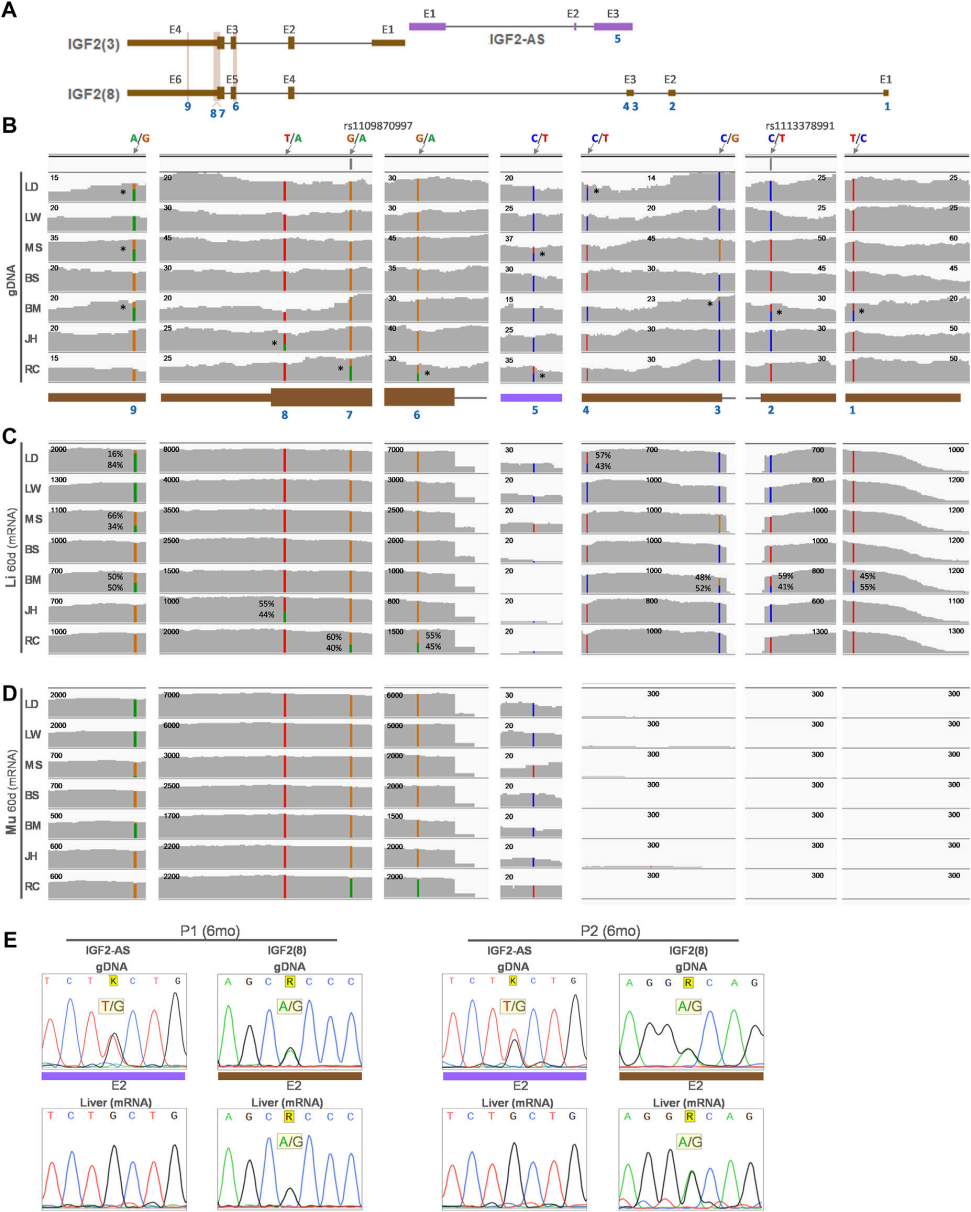
Allelic expression of the *IGF2* transcripts from the liver and skeletal muscle of 60-day-old pigs from 9 breeds. **(A)** Predominant porcine *IGF2* transcripts in liver (Li) and skeletal muscle (Mu) from Figure 4 are displayed along with an antisense transcript (*IGF2-AS*). **(B)** Location of SNPs on genomic DNA (gDNA) analyzed using WGS (PRJNA309108) are denoted with blue numbers. Reference and alternative alleles are marked in the format of ref/alt (e.g., A/G). If present, the reference SNP ID (rs ID) is shown (e.g., rs1109870997). Heterozygous alleles are denoted with stars (*) on the right side of SNPs in the genomic DNA. Read coverages of RNA-seq (GSE77776) from the same pigs as WGS are displayed for liver **(C)** and skeletal muscle **(D)**. Numbers on the top-left corner of each read coverage denote the depth of coverage. **(E)** Sequencing of exon 2 (E2) of *IGF2(8)* and *IGF2-AS* for genomic DNA and cDNA from the liver tissues of two 6-month-old (6 months) Berkshire pigs (P1 and 2). LW, Large White; LD, Landrace; MS, Meishan; BS, Berkshire; BM, Bamei; JH, Jinhua; RC, Rongchang pigs.

To examine allelic expression patterns, informative (heterozygous) SNPs in the *IGF2(3)* and *IGF2(8)* transcripts and *IGF2-AS* were explored (Figures 5A,B). Nine heterozygous SNPs were found in genomic DNA (gDNA) of any of the 60-day-old pigs (Figures 5A,B and Supplementary Table S4). Among those SNPs, four heterozygous SNPs including a previously reported SNP (rs1113378991) were found for non-overlapping exons of the *IGF2(8)* transcript in Bamei (SNP1-3) and Landrace (SNP4) pigs (Figure 5B and Supplementary Table S4) and mRNA expression on those four alleles in the liver was biallelic (Figure 5C and Supplementary Table S4), indicating biallelic expression of the porcine *IGF2(8)* transcript. One heterozygous SNP for *IGF2-AS* (SNP5) in Meishan and Rongchang pigs (Figure 5B and Supplementary Table S4) was expressed monoallelically in both the liver and skeletal muscle, although the mRNA expression level was low (Figures 5C,D and Supplementary Table S4). Four other SNPs (SNP6-9) including a previously reported SNP (rs1109870997) were found in overlapping exons of the *IGF2(3)* and *IGF2(8)* transcripts, and informative SNPs were not found in the non-overlapping exon (E1) of *IGF2(3)* (Figures 5A,B and Supplementary Table S4). In the liver of Rongchang, Jinhua, and Bamei pigs which expressed exclusively the *IGF2(8)* transcript (Figure 4), SNP6 and 7 in Rongchang, SNP8 in Jinhua, and SNP9 in Bamei pigs were biallelically expressed (Figure 5C and Supplementary Table S4). In the liver of Meishan pigs which expressed both the *IGF2(3)* and *IGF2(8)* transcripts (Figure 4), SNP9 showed a decreased biallelic tendency due possibly to monoallelic expression of *IGF2(3)* and biallelic expression of *IGF2(8)* (Figure 5C and Supplementary Table S4). In the liver of Landrace pigs which expressed the *IGF2(3)* transcript predominantly and the *IGF2(8)* transcript at a low level (Figure 4), SNP9 showed a monoallelic tendency due possibly to substantially higher monoallelic expression of the *IGF2(3)* transcript than biallelic expression of the *IGF2(8)* transcript (Figure 5C and Supplementary Table S4). These allelic changes were also found using another dataset which showed a biallelic tendency or a decreased biallelic tendency (Supplementary Figure S4B). In the skeletal muscle, these Rongchang, Jinhua, Bamei, Meishan, and Landrace pigs expressed the *IGF2(3)* transcript predominantly (Figure 4), and corresponding expression of SNP6-9 tended to be monoallelic (Figure 5D and Supplementary Table S4). In the liver of adult Berkshire pigs, biallelic expression of the *IGF2(8)* transcript and monoallelic expression of *IGF2-AS* were confirmed (Figure 5E). Taken together, it suggests that allelic expression patterns were different in between the liver and muscle, in addition to the difference of the expressed form of *IGF2* transcripts.

### 3.5 *IGF2* Expression in Humans is Developmentally Regulated and Bi- or Mono-Allelic Expression Patterns Are Tissue-Specific

**FIGURE 6:**
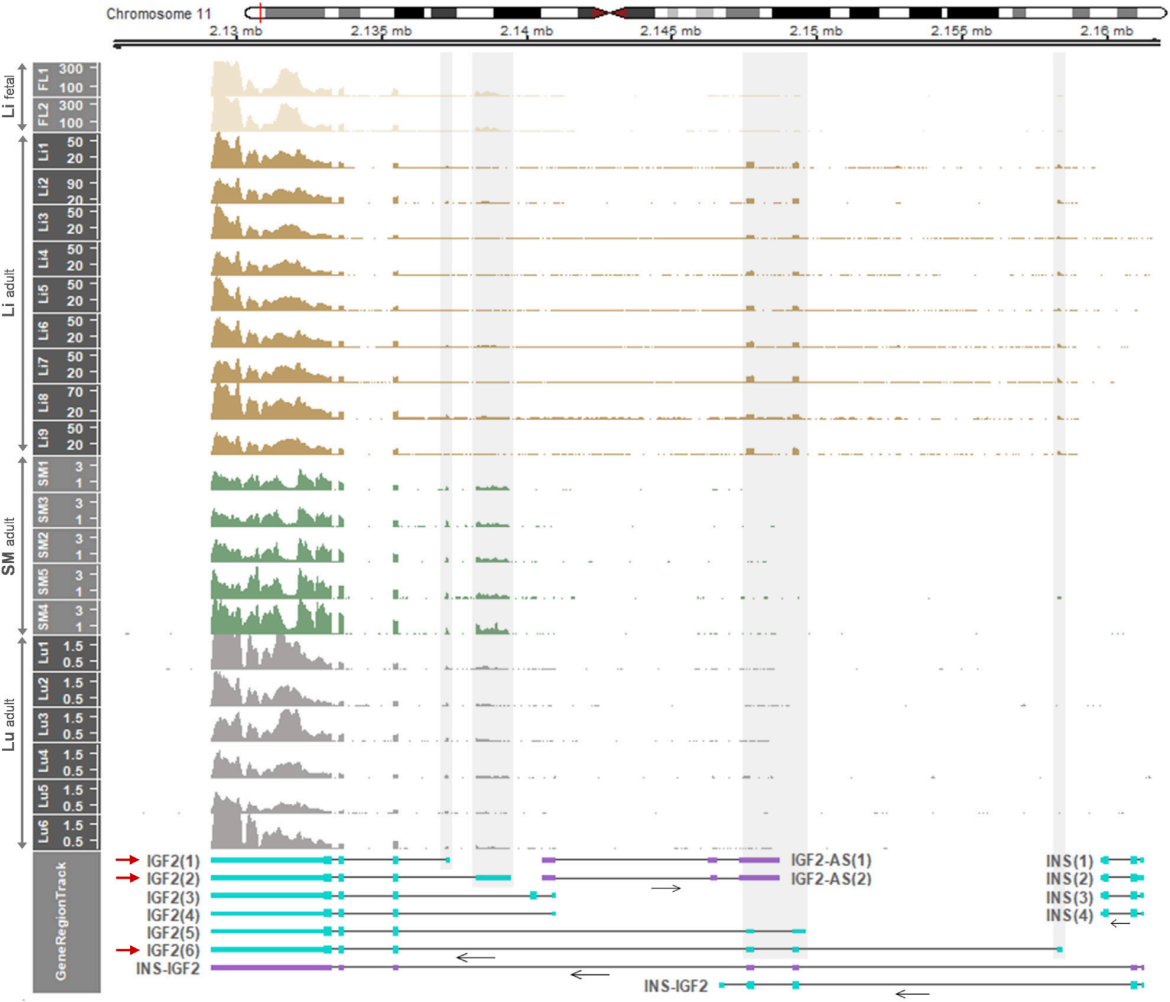
Temporal and spatial differences of expressed *IGF2* transcripts in the human. Displayed are mRNA expression of the *IGF2* gene in the human fetal liver (Li), adult liver (Li), adult smooth muscle (SM), and adult lung (Lu). RNA-seq data were retrieved from datasets: GSE63634 for fetal liver, hum0158.v2 for normal adult liver, SRP166862 for normal adult smooth muscle, and PRJNA395106 for normal adult lung. Read coverages are represented as TPM values on the y-axis. In GeneRegionTrack, transcripts of *IGF2*, *INS-IGF2* fusion, *IGF2-AS*, and *INS* genes located in the 0.0365-Mb (36.5-Kb) region (chr11:2,125,500-2,162,000) are displayed by either light blue boxes [tall, translated regions; short, untranslated regions (UTRs)] for protein-coding or purple boxes for non-coding. Horizontal arrows under transcripts indicate the direction of transcription. Grey shades overlay non-overlapping exons with high read coverages corresponding to predominant transcripts (*IGF2(1)*, *IGF2(2)* and *IGF2(6)*) with red arrows. Human gene transcripts include *IGF2(1)*, NM_001291861.3; *IGF2(2)*, NM_000612.6; *IGF2(3)*, NM_001127598.3; *IGF2(4)*, NM_001291862.3; *IGF2(5)*, NM_001007139.5; *IGF2-AS(1)*, NR_028043.2; *IGF2-AS(2)*, NR_133657.1; *INS(1)*, NM_001185097.2; *INS(2)*, NM_001185098.2; *INS(3)*, NM_001291897.2; *INS(4)*, NM_000207.3.

**FIGURE 7:**
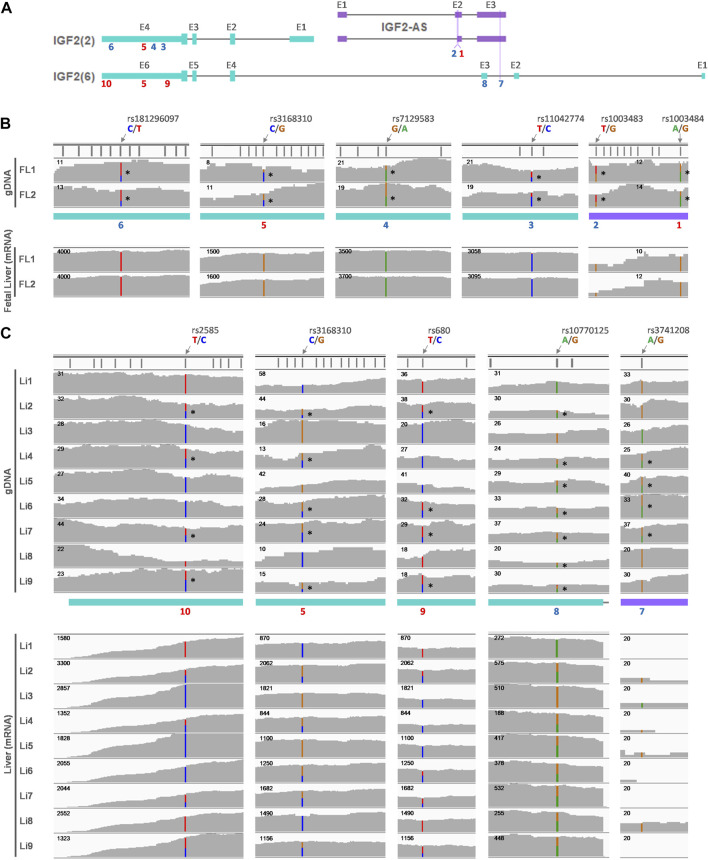
Allelic expression of *IGF2* in fetal and adult human liver. **(A)** Predominant *IGF2* transcripts in either the fetal liver (FL) (*IGF2(2)*) or adult liver (Li) (*IGF2(6)*) are displayed along with antisense transcripts (*IGF2-AS*). SNP locations are marked with numbers on exons (E) and numbers in red (5, 9, and 10) indicate the same SNPs as in Figure 7. **(B)** Heterozygous alleles in the gDNA were identified using H3K4me1 ChIP-seq data retried from GSE63634 for fetal liver at 12 weeks of gestation and are denoted with stars (*) on the right side of SNPs. Corresponding sites were displayed below for fetal liver mRNA expression in individual-matched RNA-seq. **(C)** WGS data obtained from hum0158.v2 for normal adult liver, and heterozygous alleles are marked with stars (*) on the right side of SNPs. Corresponding mRNA expression in the adult liver derived from RNA-seq from the same dataset is shown below.

For comparison with pigs, the *IGF2* gene expression in the fetal and adult liver and other tissues of the human was examined and also allelic expression in those tissues was further investigated. Based on RNA-seq read coverages, when compared with other tissues, expression of *IGF2* and *H19* was relatively high in the liver and muscle, respectively, while expression of both *IGF2* and *H19* was low in other tissues including the brain, lung, colon, and stomach (Supplementary Figure S6A). The *IGF2(2)* transcript [the orthologous transcript of porcine *IGF2(3)*] was expressed in the fetal liver and the *IGF2(6)* transcript [the orthologous transcript of porcine *IGF2(8)*] was expressed in the adult liver (Figure 6 and Supplementary Figures S6, S7), which are comparable to the findings of the predominant expression of *IGF2(3)* and *IGF2(8)* in fetal and adult livers, respectively, in pigs. Both the *IGF2(1)* and *IGF2(2)* transcripts were expressed in the adult smooth muscle and lung (Figure 6). The overall expression levels tended to be high to low in the order of fetal liver, adult liver, adult smooth muscle, and adult lung. Expression of *INS-IGF2* fusion transcripts and *INS* transcripts was not detectable (Figure 6), while expression of *IGF2-AS* was low but detectable in the fetal liver and decreased in postnatal stages of both pigs and humans (Figure 7B and Supplementary Figure S8). Total expression of *IGF2* was significantly higher in the liver than in skeletal muscle, and total expression of *H19* tended to be higher in skeletal muscle than in the liver (Supplementary Figure S6B), and these patterns in adult humans were similar to those of the adult pigs (Supplementary Figure S2B).

Allelic expression was further explored for the fetal and adult liver of the human, regarding informative SNPs in the expressed *IGF2(2)* and *IGF2(6)* transcripts and *IGF2-AS* (Figure 7A). The *IGF2(2)* and *IGF2-AS* transcripts were expressed in the fetal liver (Figures 7A,B), and six informative SNPs on gDNA were found (Figure 7B and Supplementary Table S5). All of those six SNPs were monoallelically expressed in mRNA of the fetal liver (Figure 7B and Supplementary Table S5). The *IGF2(6)* transcript was expressed in the adult liver and with a very low degree for *IGF2-AS* expression (Figures 7A,C). The same SNP5 between the fetal and adult liver was heterozygous in the adult liver from individual 2, 4, 6, 7, and 9 (Figure 7C top panel and Supplementary Table S5) and tended to be expressed biallelically (Figure 7C bottom panel and Supplementary Table S5), suggesting biallelic expression of *IGF2(6)* unlikely to monoallelic expression of *IGF2(2)* in the fetal liver (Figure 7B and Supplementary Table S5). Also, in the adult liver, SNP8-10 were found to be heterozygous in some individuals (SNP8: 2 and 4-9, SNP9: 2, 6, 7, and 9, SNP10: 2, 4, 7, and 9) and corresponding mRNA expression of *IGF2(6)* tended to be biallelic (Figure 7C and Supplementary Table S5). The SNP7 was found on *IGF2-AS* but the expression was too low to determine its allelic expression (Figures 7A,C and Supplementary Table S5) and therefore, its allelic expression could not be determined.

**FIGURE 8:**
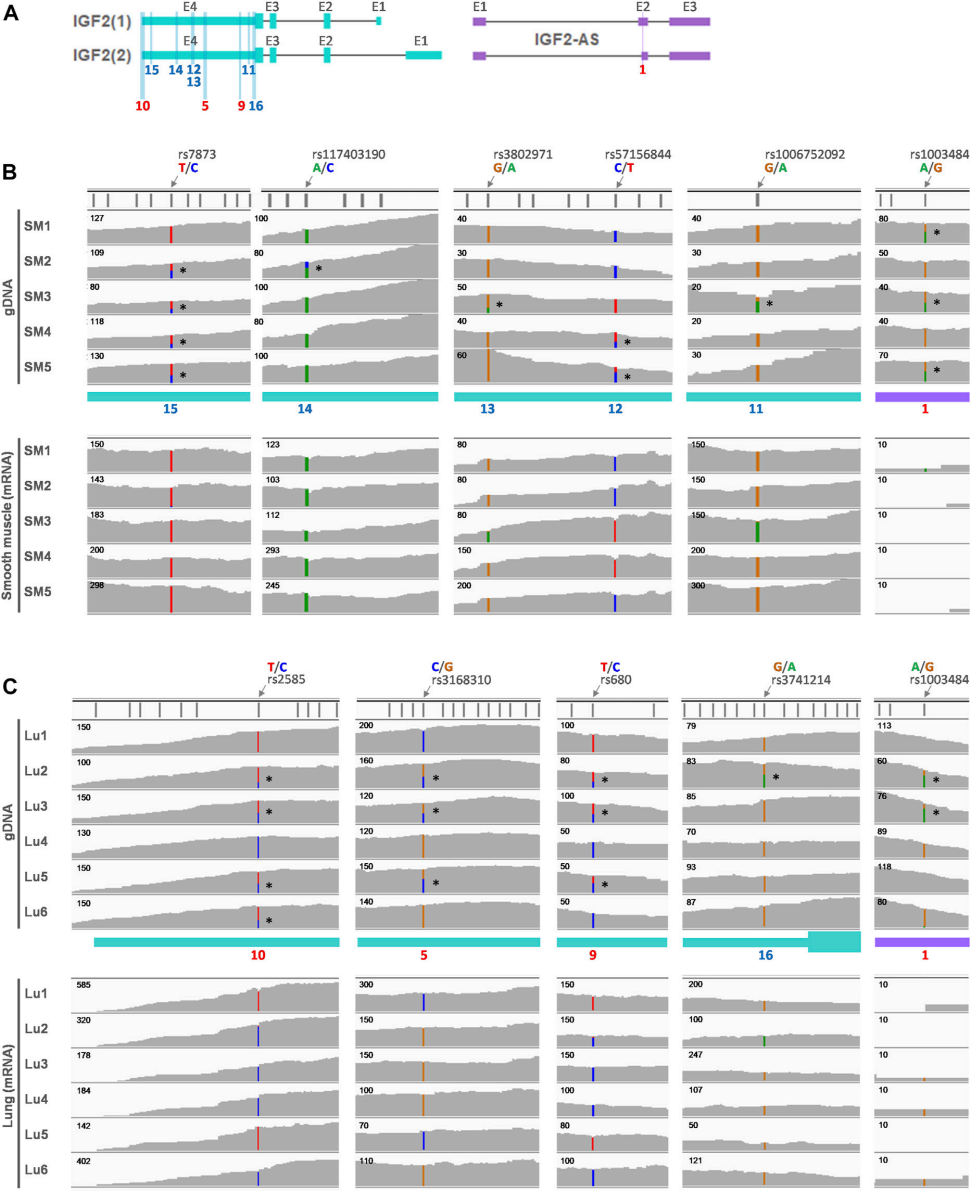
Allelic expression of *IGF2* in adult human smooth muscle and lung. **(A)** Predominant *IGF2* transcripts (*IGF2(1)* and *IGF2(2)*) in both adult smooth muscle (SM) and adult lung (Lu) are displayed along with antisense transcripts (*IGF2-AS*). SNP locations are marked with numbers on exons (E) and numbers in red (5, 9, and 10) indicate the same SNPs as in Figure 7. **(B)** Whole-exome sequencing (WES) data (SRP163897) generated using adult smooth muscle were retrieved, and heterozygous alleles are marked on the gDNA. Corresponding matched RNA-seq (SRP166862) were processed and allelic expression is displayed below. **(C)** Heterozygous alleles were identified using WES data (PRJNA395106) of the human adult lung and marked with *. Corresponding matched RNA-seq from the same dataset (PRJNA395106) were used to show allelic expression.

Moreover, in smooth muscle and lungs, informative SNPs on *IGF2(1)*, *IGF2(2)*, and *IGF2-AS* were explored in gDNA (Figure 8A) and their allelic expression was examined. In smooth muscle, both *IGF2(1)* and *IGF2(2)* were expressed, and SNP11-15 were heterozygous in gDNA (Figures 8A,B and Supplementary Table S6). Allelic expression of SNP11-15 tended to be monoallelic (Figure 8B bottom panel and Supplementary Table S6), indicating monoallelic expression of *IGF2(1)* and *IGF2(2)*. The SNP1 was heterozygous in smooth muscle, but the mRNA expression was very low (Figures 8A,B and Supplementary Table S6). In the lungs, both *IGF2(1)* and *IGF2(2)* were expressed, and the same SNP5, 9, and 10 as in the liver were found to be heterozygous (Figures 8A,C and Supplementary Table S6). Unlike biallelic expression in the liver, expression on SNP5, 9, and 10 was monoallelic in the lungs (Figure 8C bottom panel and Supplementary Table S6). In addition, SNP16 was found to be heterozygous in individual 2, and its expression tended to be monoallelic in the lungs (Figures 8A,C and Supplementary Table S6). Regarding SNP1, the mRNA expression of *IGF2-AS* was very low in the lungs (Figures 8A,C and Supplementary Table S6).

Overall, in the human, *IGF2(1)* and/or *IGF2(2)* were expressed in the fetal liver, adult smooth muscle, and adult lung, and the expression tended to be monoallelic, whereas *IGF2(6)* was expressed in the adult liver and the expression tended to be biallelic. In addition, the *IGF2-AS* expression was very low, except in the fetal liver where the expression tended to be monoallelic.

### 3.6 Topologically Associating Domains in the *H19/IGF2* Imprinted Cluster and Schematic Models Represent Insulation and Imprinting Boundaries

**FIGURE 9:**
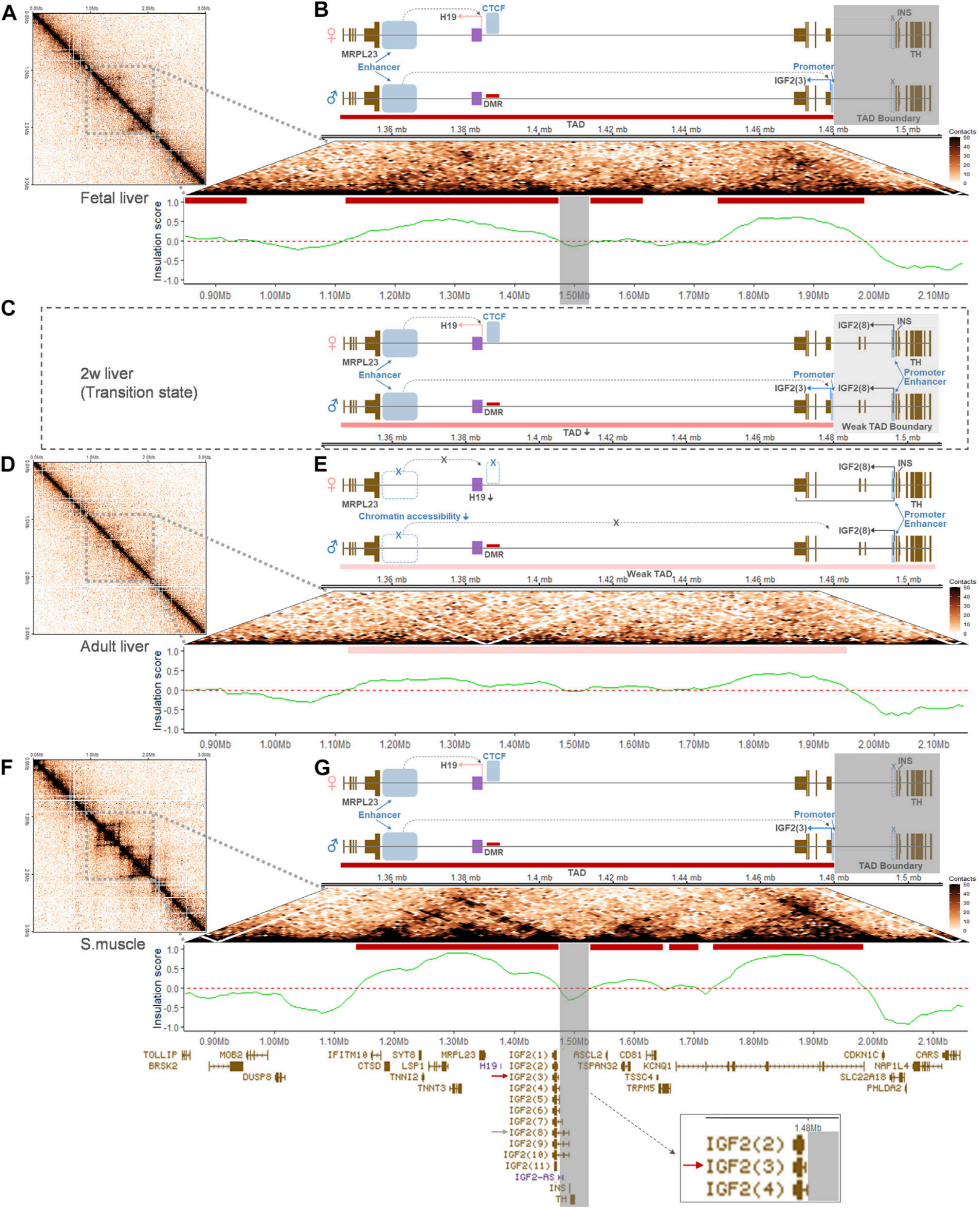
Chromatin interactions and schematic models of gene regulation at the porcine *H19/IGF2* locus. For the chromosomal region (chr2:0-3000000) containing the *H19/IGF2* locus, two-dimensional heatmaps of Hi-C contact matrices were generated at a 10-kb resolution using the GENOVA R-package. Corresponding truncated pyramid plots zoomed in the locus. TADs were identified based on insulation scores and are indicated with red bars. **(A,B)** In the fetal liver, a TAD boundary is indicated with the grey perpendicular shades and a schematic diagram displays the long-range enhancer-promoter communication for paternal *IGF2(3)* expression within the TAD. Active regulatory elements are denoted with blue rectangles. In the maternal allele, CTCF binds to the hypomethylated region and expression of *H19* is promoted by an active enhancer. **(C)** A proposed schematic model of less chromatin interaction and week TAD boundary is shown for a transition state around 2 weeks (2w) for the porcine liver. Both paternal *IGF2(3)* expression and biallelic *IGF(8)* expression are indicated. **(D,E)** In the adult liver, a week TAD throughout the region is marked with pink bars. Also, low chromatin accessibility in the downstream of *H19* and altered gene regulation are represented in the diagram, along with biallelic *IGF(8)* expression indicated by black bent arrows on both alleles. **(F,G)** In skeletal muscle, the grey perpendicular shades denote a TAD boundary and paternal *IGF2(3)* expression is indicated, similarly to the ones in the fetal liver **(A,B)**. Gene transcripts expressed throughout the region are displayed in the bottom track. A red arrow marks the predominant *IGF2* isoform, *IGF2(3)*, in the fetal liver **(B)** and skeletal muscle **(G)**, and a grey arrow points at the long-form, *IGF2(8)*, which is expressed predominantly in the adult liver **(E)**. Both *IGF2(3)* and *IGF2(8)* are expressed in the proposed transition state of pigs **(C)**.

Hi-C datasets were analyzed to identify topologically associating domains (TADs) and TAD boundaries within and adjacent to the *H19/IGF2* locus. To determine whether Hi-C data from replicates of the liver and skeletal muscle can be merged, concordance of the data was estimated using GenomeDISCO (Ursu et al., 2018). For both fetal and adult livers, concordance scores for all pairwise comparisons of Hi-C matrices in multiple resolutions passed a threshold of 0.8 while also passing the recommended threshold of 0.8 at a 50-kb resolution (Ursu et al., 2018), although the scores tended to decrease in higher resolutions (Supplementary Figure S9). In addition, smoothed matrices produced from GenomeDISCO procedures displayed similarities within the tissue groups, but not between the groups (Supplementary Figure S10–S15). For skeletal muscle of 2-week-old pigs, concordance scores from pairwise comparison also passed the threshold and smoothed matrices were different from those of fetal and adult livers (Supplementary Figures S16, S17). Total number of trimmed paired-end reads of each group were similar: ∼2.35 billion for fetal liver, ∼2.90 billion for the liver, and ∼2.24 billion for skeletal muscle (Supplementary Table S7). Based on high concordance and comparable amount of the data, matrices of three, three, and two replicates for each group (fetal liver, adult liver, and skeletal muscle), respectively, were merged to increase resolution. After merging, Hi-C matrices at a 10-kb resolution were visualized using two-dimensional heatmaps, and the matrices of the fetal liver and skeletal muscle exhibited stronger contact interactions between approximately 1.0 and 2.0 Mb of pig chromosome 2 than that of the adult liver (Figure 9). In the fetal liver and skeletal muscle, boundaries between TADs (i.e., TAD boundaries) encompassing the locus where the first exon of *IGF2(8)* starts, but not the first exon of *IGF2(3)*, was found (Figures 9A,F). In addition, muscle from fetal pigs contained a TAD boundary at the locus containing the first exon of *IGF2(8)* which was not overlapped with the first exon of *IGF2(3)* (Supplementary Figure S18). In contrast, in the adult liver, a week TAD throughout the region based on insulation scores was revealed, and TAD boundaries were absent at the *H19/IGF2* locus (Figure 9D). We expected lowered chromatin interaction in the liver at a transition state based on the enriched regulatory elements and gene expression patterns (Figure 3), but Hi-C data were absent (Figure 9C). In schematic diagrams, we propose chromatin reorganization in the liver that can weaken TAD and TAD boundaries and alter gene regulation, resulting in conversion of monoallelic expression of *IGF2(3)* in the fetal liver (Figure 9B) *via* both monoallelic expression of *IGF2(3)* and biallelic expression of *IGF2(8)* in the neonatal liver (Figure 9C) to biallelic expression of *IGF2(8)* in the adult liver (Figure 9E). In skeletal muscle, consistent *IGF2(3)* expression throughout the development and similar chromatin interaction frequencies between pre- and post-natal stages suggested robust imprinted gene regulation underlying monoallelic expression of the *IGF2* transcript (Figure 9G).

## 4 Discussion

In this study, we present comprehensive imprinting status of the conserved paternally imprinted *H19/IGF2* cluster including developmentally regulated and tissue-specific allelic *IGF2* gene expression in the pig and human. By comparing methylome of parthenogenetic (diploid uni-maternal) embryos with bi-parental control embryos, while reducing genetic variability with triplicates of each sample, the porcine *H19* DMR was identified. Previously, the paternal methylation imprint on the *H19* germline DMR, which is fully methylated in sperm and unmethylated in oocytes, was reported in pigs in the form of a group of three DMRs (Park et al., 2009). On the paternal allele of the *H19* DMR, however, demethylation temporarily occurs and then it is remethylated by the morula stage (Park et al., 2009). In addition, differential expression of *IGF2* between androgenetic, parthenogenetic, and *in vitro* fertilized control embryos was previously observed from the blastocyst stage around day 10 (Park et al., 2011). Because, in this study, parthenogenetic and control embryos were recovered later at embryonic day 21at which the dynamic methylation changes were passed, the detected *H19* DMR could be consistent with the germline DMR between sperm and oocytes. The recovery day 21 was also before morphological degeneration of parthenogenetic embryos occurs at around day 30–35 (Bischoff et al., 2009; Hwang et al., 2020) so that we could prevent confounding effects other than genetic effects. On the other hand, putative *IGF2* DMRs, which were hypermethylated in sperm DNA of Swiss Landrace and Swiss Large White (Giannini and Braunschweig, 2009), were not found in the current study possibly due to breed-specific effects on DNA methylation as described previously (Hwang et al., 2020).

Integrative analyses of ATAC-seq, ChIP-seq, and RNA-seq datasets provide effective strategies to precisely and spatiotemporally elucidate epigenetic regulatory elements and their genetic variations that affect gene expression (Buenrostro et al., 2013; Lara-Astiaso et al., 2014; Floc’hlay et al., 2020). While variations on the regulatory DNA are often buffered and compensated by other regulatory elements so that redundant regulatory signals might be present in the *H19/IGF2* locus (Floc’hlay et al., 2020), clear distinctions of regulatory layers between the liver and muscle tissues were identified (Figure 2). As the ATAC signals are substantially correlated with H3K27ac (Lara-Astiaso et al., 2014), co-occurrence of ATAC and H3K27ac downstream of *H19* suggested that active enhancers were established from a poised state concomitantly with formation of open chromatin sites upon developmental and signaling cues (Creyghton et al., 2010). Interestingly, this activation of enhancers occurred in skeletal muscle, but not in the liver, of 6-month-old pigs, which leads to recruitment of tissue-specific transcription factors and drives tissue-specific gene expression (Ong and Corces, 2011). In addition, in eukaryotes, H3K4me3 is associated with transcriptional activation on active promoters and typically restricted to narrow regions at the 5′ end of the gene body (Santos-Rosa et al., 2002; Schneider et al., 2004; Pena et al., 2006; Wysocka et al., 2006). H3K4me3 marks overlapping the CTCF signal immediately upstream of *H19* might represent the active *H19* promoter regulated by binding of the transcription factor CTCF at close range. Also, H3K4me3 marks overlapping the promoter regions of *IGF2* transcripts [i.e., *IGF2(3)* and *IGF2(8)*] might represent transcriptionally active *IGF2* promoters. At both proximal and distal regions of TSSs the H3K27ac signal can be found (Creyghton et al., 2010), and thus overlaps of H3K27ac with H3K4me3 at close proximity to the promoter regions of *IGF2* transcripts might represent both active enhancers and promoters. We found that these overlaps of H3K27ac and H3K4me3 were present near the first exons of both *IGF2(3)* and *IGF2(8)* transcripts in the liver of 2-week-old pigs, indicating distinct gene regulation during early post-natal liver development.

Although many imprinted genes have been studied in the fetal stage because of their relevance to fetal growth (Peters, 2014; Tian, 2014; Tucci et al., 2019), the current study revealed that the expressed transcript isoform of *IGF2* in muscle tissues might be stably maintained during development and its monoallelic expression was identified in post-natal stages suggesting its role in mature muscle. In the liver of fetal pigs, there was a lack of informative SNPs, but our parthenogenesis studies with whole embryos showed paternal monoallelic expression in the embryonic stage (Figure 1B). In the human, it has been reported that *IGF2* gene transcription is driven by multiple promoters in fetal and non-hepatic adult tissues (Holthuizen et al., 1993; Monk et al., 2006), but we showed that the major form in these tissues of humans was *IGF2(2)* which is orthologous to porcine *IGF2(3)*. On the other hand, the liver-specific promoter (P1) drives *IGF2* gene transcription in the adult liver (Holthuizen et al., 1993; Monk et al., 2006). The corresponding adult liver-specific transcript is not currently annotated in the NCBI Gene database (https://www.ncbi.nlm. nih.gov/gene/3481), but we revealed the expression of adult liver-specific *IGF2(6)* (Supplementary Figure S7) which was orthologous to the porcine long-form transcript [*IGF2(8)*]. The *IGF2* transcripts from human fetal tissues including the liver is paternally imprinted and monoallelic, but expression becomes biallelic in the adult liver (Kalscheuer et al., 1993). In addition to this biallelic conversion, we revealed that a relatively high *IGF2* expression occurred in the adult human liver compared to other analyzed tissues where monoallelic expression remains (Figure 6 and Supplementary Figure S6). Our analyses using pigs support the biallelic conversion and alternative promoter usage that might occur gradually at post-natal ages while ages for initiation of the conversion might vary (Figure 4). These allelic expression patterns were verified based on individual-matched genomic DNA sequence data from WGS and mRNA sequence data from RNA-seq in both pigs and humans (Figures 5, 7, and 8) using informative SNPs found on genomic DNA that served as markers to confirm allelic imbalance of mRNA expression (Castel et al., 2015; Ahn et al., 2021b; a). We primarily examined SNPs in non-overlapping exons to identify and analyze allelic expression at the isoform level while there is a previous study relied on a marker in the last overlapping exon (Braunschweig et al., 2011). In contrast to the *INS-IGF2* read-through script whose expression is spatially regulated in pancreatic islets (Jian and Felsenfeld, 2021), the expression of *IGF2-AS* transcript has been shown to be developmentally regulated as its imprinted paternal expression is relatively high in fetal stages and decreased in adults in both pigs and humans (Okutsu et al., 2000; Braunschweig et al., 2004). Moreover, the notion that the expression of *IGF2-AS* in fetal stages interferes with overlapping *IGF2* (Braunschweig et al., 2004) might be supported by our findings: relatively high expression of *IGF2-AS* in the fetal liver coincided with negligible expression of the long-forms [porcine *IGF2(8)* and human *IGF2(6)*] and low expression of *IGF2-AS* in adults coincided with high expression of the long-forms. However, it is also expected that the antisense role of *IGF2-AS* might be limited in normal tissues due to its low expression compared with high expression of *IGF2,* although increased expression of *IGF2-AS* has been reported in Wilms tumors (Okutsu et al., 2000). Rather, the biallelic conversion in the liver might ensue changes in chromatin structure and regulatory elements as well as antisense expression as discussed below.

The differences in chromatin interaction between fetal and adult livers (and also between skeletal muscle and adult liver) suggested not only transcript conversion, but also chromatin remodeling might occur toward changes in gene regulatory elements and reduce long-range enhancer-promoter communication in the adult liver (Figure 9 and Supplementary Figure S18). In particular, hemi-methylation at the *H19* DMR in the adult liver was reported indicating maintenance of the imprint (Braunschweig et al., 2011); however, compared to the fetal liver, chromatin interaction indicated by the self-interacting TADs became weaker in the adult liver of pigs. This lower interaction might be related to less activity of the distal enhancer for the long-range communication, and removal of TAD boundaries might lead to use of the proximal enhancer for the long *IGF2* transcript in the liver. Also, a linkage between less *H19* expression indicating the weak distal enhancer and expression of the biallelic *IGF2* transcript (Ohlsson et al., 1994) was consistently observed in the livers of both pigs and humans (Supplementary Figures S2 and S6). Additionally, in between the fetal and adult stages, there might be a transition state in the liver that is characterized by co-existence of TADs and a weak TAD boundary which is permissive to the proximal enhancer activity (Gong et al., 2018). In contrast, in skeletal muscle, TADs and TAD boundaries in fetal stages appeared to maintain in post-natal ages. Whether these TADs and TAD boundaries are variable at the single cell level will need to be further investigated (Farabella and Marti-Renom, 2020; Luppino et al., 2020), but existence of TADs and TAD boundaries at the porcine *IGF2* locus was evident. Their significant changes and remodeling in the liver might contribute to liver-specific modifications of *IGF2* allelic expression patterns. Our presentation of the pig Hi-C fills the gap in mammalian genomics, but unfortunately, in the human, Hi-C data from liver (GSE58752) and muscle (GSE87112) tissues that we processed displayed a very low resolution for this relatively narrow range of the *H19/IGF2* locus. Based on our current study that advances our understanding on tissue-specific genomic imprinting in the *H19/IGF2* cluster, studies on other animal species using multi-omics data can further comparatively delineate the *H19/IGF2* locus. Also, gene annotations for porcine *H19* and *IGF2-AS* and human *IGF2(6)* need to be updated due to their lack in the NCBI Gene database (www.ncbi.nlm.nih.gov/gene). In this study, based on previous studies reported *H19* and *IGF2-AS* expression status in pigs (Li et al., 2008; Braunschweig et al., 2011) and *IGF2(6)* in humans (Holthuizen et al., 1993; Monk et al., 2006) as well as our alignment and sequencing results, these genes were analyzed to present the complete landscape of genomic imprinting.

## 5 Conclusion

Our integrative omics analyses of genome, epigenome, and transcriptome revealed a comprehensive imprinting status at the *H19/IGF2* locus in pigs in comparison with humans. The porcine *H19*/*IGF2* imprinting cluster represented a long-term influence of genomic imprinting in muscle tissues but not in the liver which might be similar to that of the orthologous human gene cluster. To the best of our knowledge, this is the first study that describes relatedness between mono- to biallelic conversion of *IGF2* and alternative promoter usage in reorganized chromatin in the liver of adult pigs. The current approaches can be applied in cross-tissue and cross-species analyses to elucidate epigenetic mechanisms that underlie tissue growth and development.

## Data Availability

The datasets generated for this study can be found in the NCBI GEO repository under accession number GSE195528. The publicly available datasets analyzed for this study are summarized in Materials and Methods and listed in Supplementary Table S8.
