# Supplementary material for: Loss of Monoallelic Expression of IGF2 in the Adult Liver Via Alternative Promoter Usage and Chromatin Reorganization
Source: Front Genet. 2022 Jul 22;13:920641. doi: 10.3389/fgene.2022.920641 (PMC9355166; doi:10.3389/fgene.2022.920641)
Supplement: Supplementary file 1 [file DataSheet1.PDF]

Supplementary Material

1 Supplementary Figures and Tables

1.1 Supplementary Figures

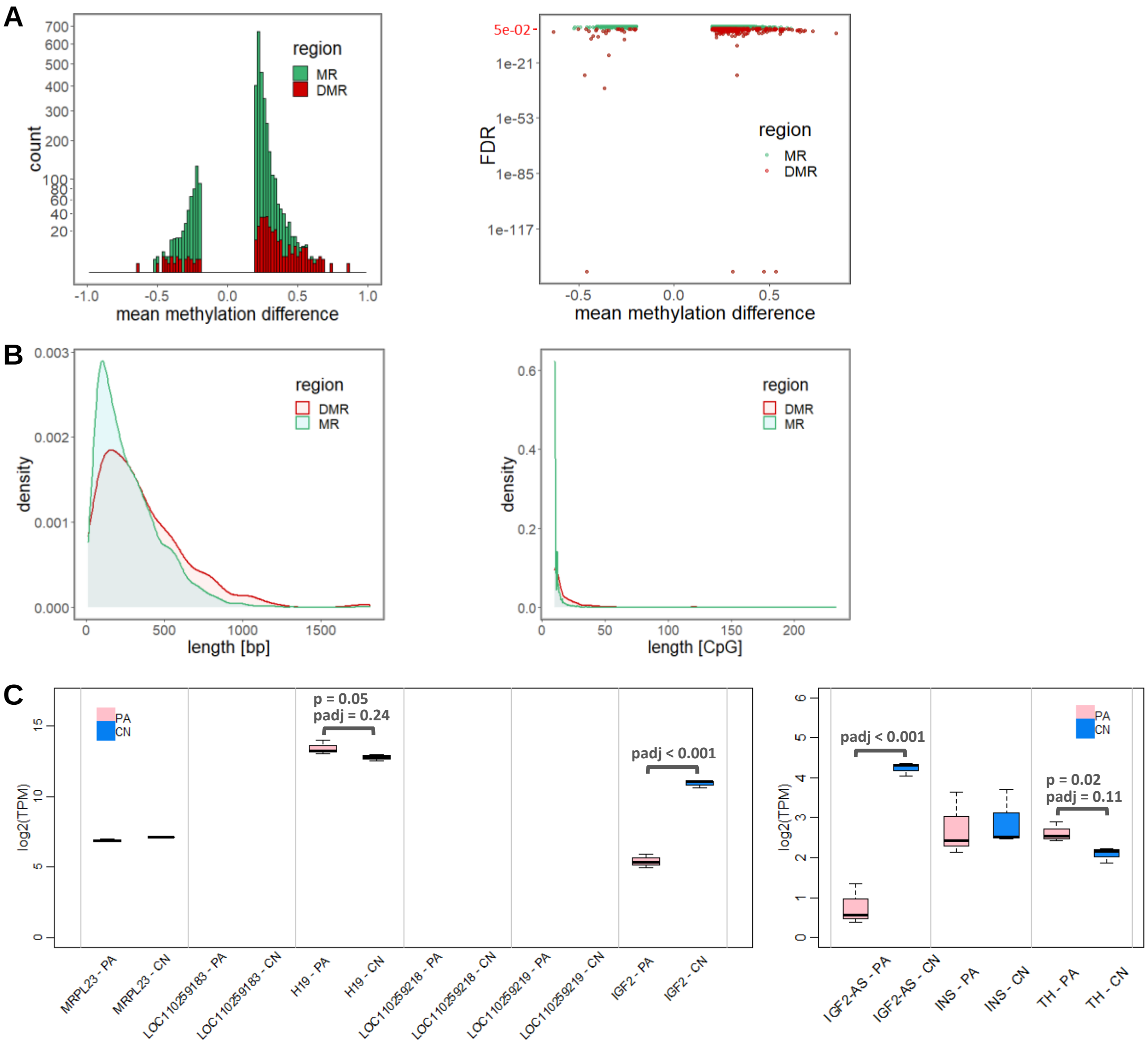

Supplementary Figure 1. Distribution of WGBS data (A-B) and differential gene expression based on RNA-seq (C) of PA and CN porcine embryos. **(A)** Methylated regions (MR) passed criteria [maximum CpG distance of 300 bp, minimum CpG number of 10, minimum mean methylation difference (PA ave – CN ave) of 0.2] and differentially methylated regions (DMR) passed the criteria and FDR < 0.05 in pig chromosome 2. Left: A histogram plotted against count (square root transformed y-axis) for DMR is overlaid on MR. Right: A dot plot of FDR displays threshold of 0.05 (5e-02) for DMR, and significant DMRs are shown towards the bottom. **(B)** Density plots (area under curve = 1) of MR and DMR lengths in base pairs (bp) (left) and CpG dinucleotide numbers (right). **(C)** Differential gene expression in between whole PA and CN embryos is indicated with adjusted p-values (padj) less than 0.05. Normalized gene expression levels in log2-transformed TPM values (y axis) within the *H19*/*IGF2* locus are based on gene-level quantification of RNA-seq data.

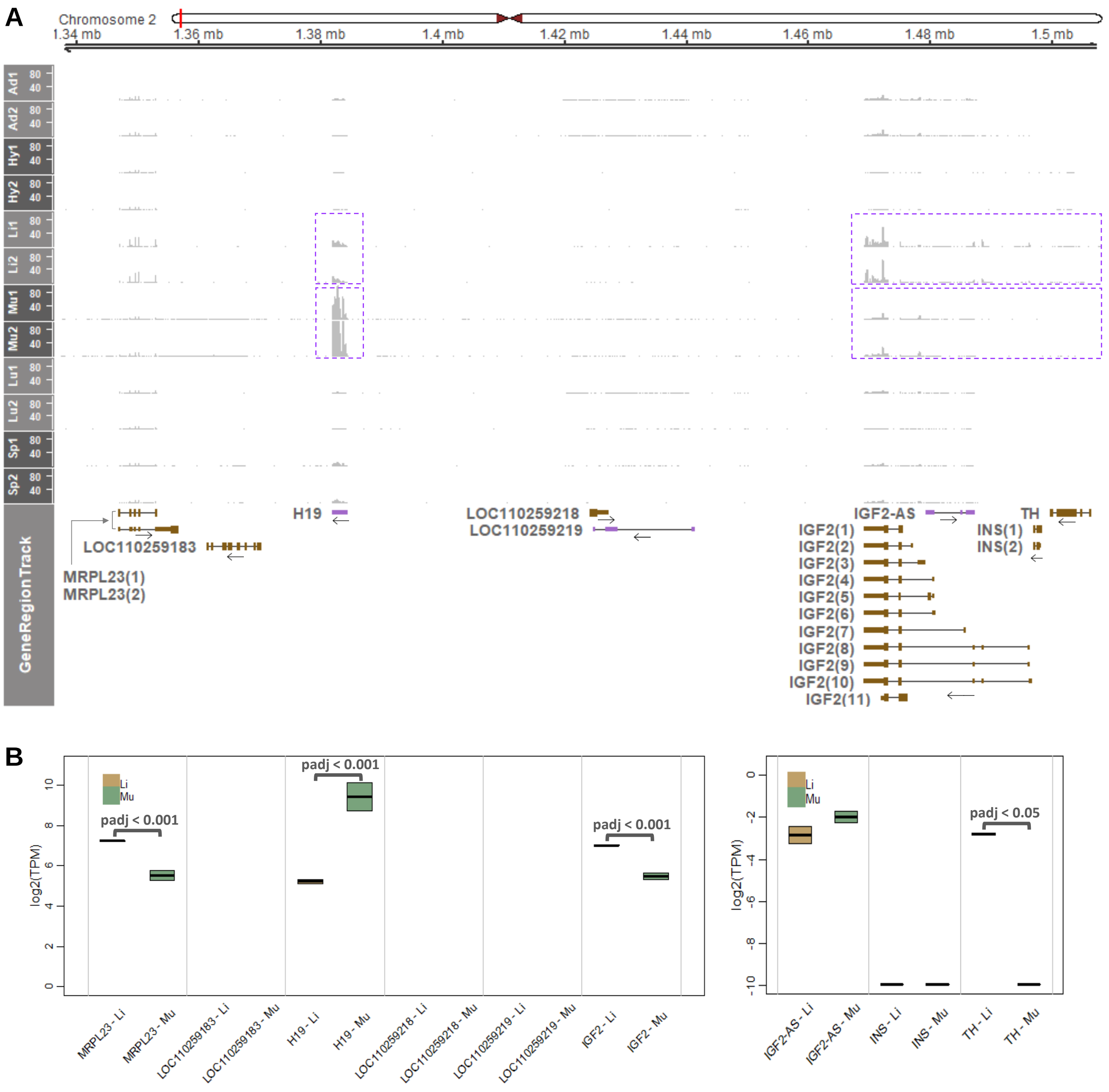

Supplementary Figure 2. Profiling of gene expression in the *H19/IGF2* locus in 6-month-old pigs. **(A)** Tissue distribution of mRNA expression based on RNA-seq read coverages. Values are represented as TPM (y-axis). Ad, adipose tissue; Hy, brain hypothalamus; Li, liver; Mu, skeletal muscle; Lu, lung; Sp, spleen. I, CpG island; GC%, percentage of CG dinucleotides. Read coverages around *H19* and *IGF2* in the liver and skeletal muscle are highlighted with dotted rectangles in purple. **(B)** Differential gene expression in between the liver and skeletal muscle of the pigs. Adjusted p-values (padj) less than 0.05 indicate statistically significant differences. Gene-level quantification on RNA-seq data (GSE158430) was performed before normalization and log2-transformed TPM values were plotted on y axis. RNA-seq run IDs are SRR12697090 and SRR12697091 (adipose), SRR12697108 and SRR12697109 (brain hypothalamus), SRR12697114 and SRR12697115 (liver), SRR12697126 and SRR12697127 (skeletal muscle), SRR12697120 and SRR12697121 (lung), and SRR12697132 and SRR12697133 (spleen).

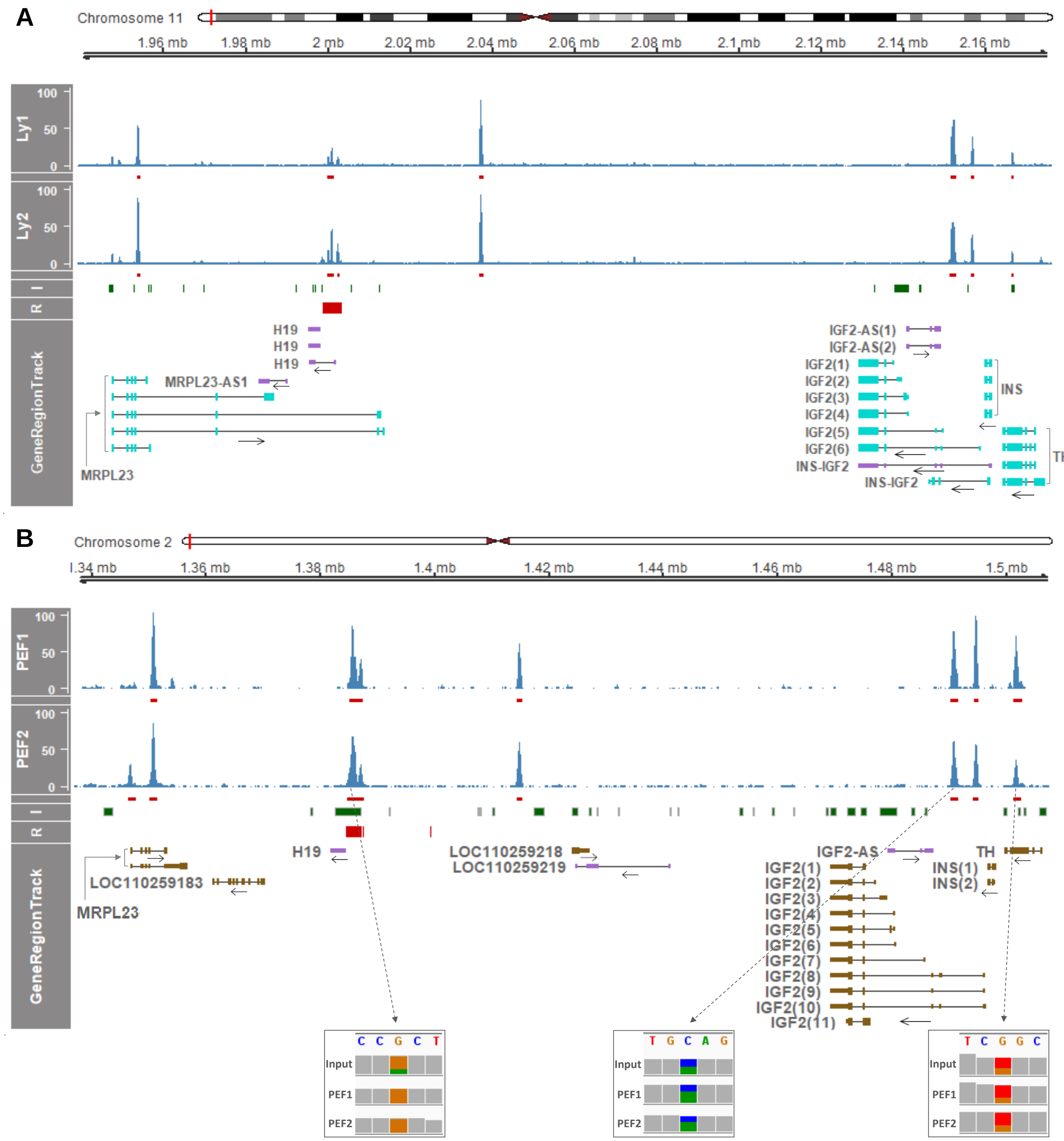

Supplementary Figure 3. CTCF enrichment in normal human lymphoblasts (Ly) (GSE155324) **(A)** and normal pig embryonic fibroblasts (PEF) (GSE153441). **(B)** presented in RPGC values in y-axis. a R track represents the human imprinting control region (ICR) retrieved from NCBI Gene database (www.ncbi.nlm.nih.gov/gene). b R track represents the porcine DMR. Red bars underscore MACS2 called peaks. I, CpG islands. In the bottom boxes, heterozygous alleles are shown in input control DNA without antibody treatment (chr2:1,385,154 (G/A), chr2:1,491,015 (C/A), and chr2:1,501,707 (G/T)). The left box indicates monoallelic precipitation via CTCF binding in PEF1 and PEF2 immediate upstream (5′) of *H19*. The middle and right boxes show biallelic precipitation via CTCF binding upstream of *IGF2* gene transcripts.

A

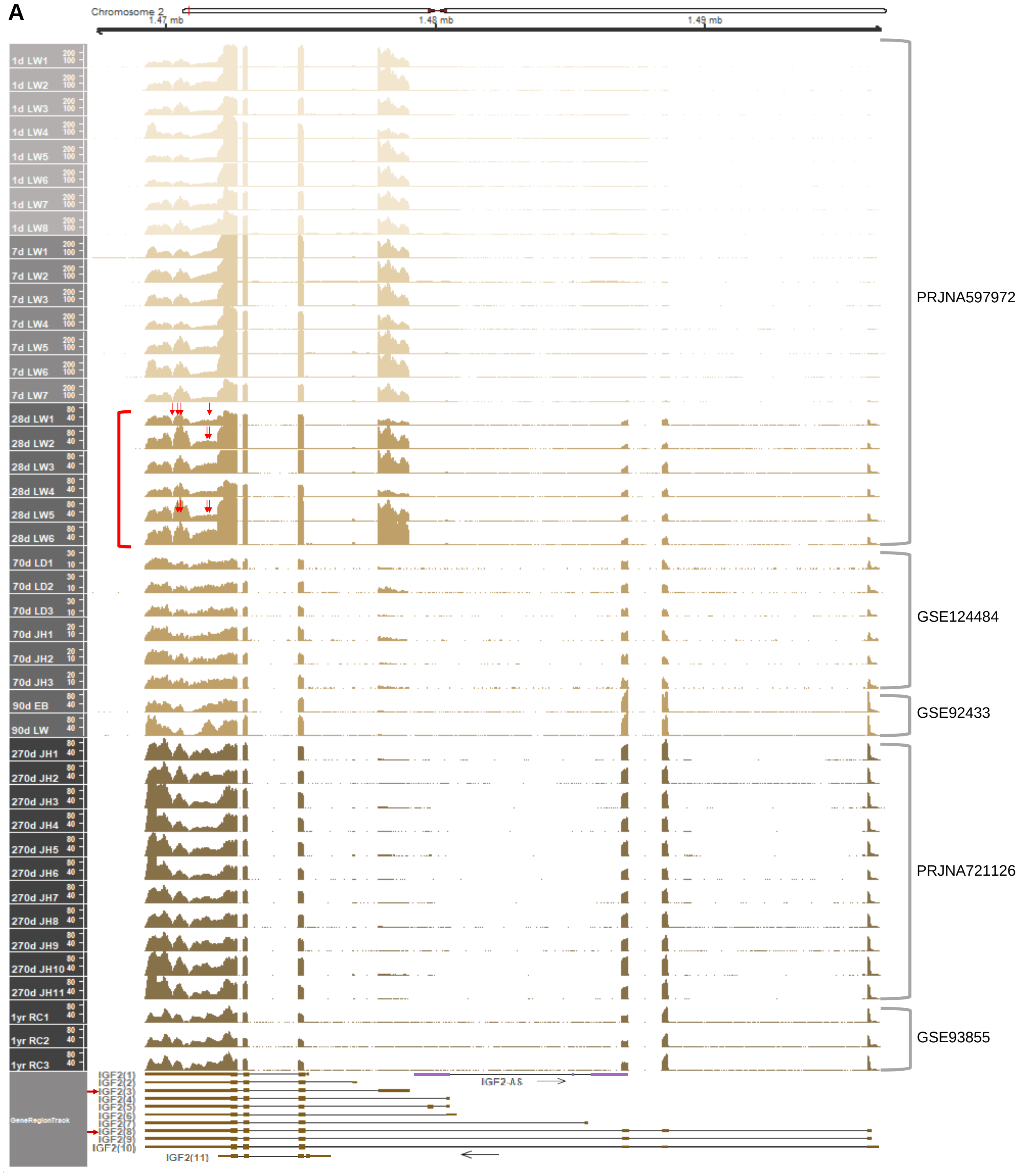
Chromosome 2
1.47 mb
1.48 mb
1.49 mb
1d LW1
1d LW2
1d LW3
1d LW4
1d LW5
1d LW6
1d LW7
1d LW8
7d LW1
7d LW2
7d LW3
7d LW4
7d LW5
7d LW6
7d LW7
28d LW1
28d LW2
28d LW3
28d LW4
28d LW5
28d LW6
70d LD1
70d LD2
70d LD3
70d JH1
70d JH2
70d JH3
90d EB
90d LW
270d JH1
270d JH2
270d JH3
270d JH4
270d JH5
270d JH6
270d JH7
270d JH8
270d JH9
270d JH10
270d JH11
1yr RC1
1yr RC2
1yr RC3
GeneRegionTrack
IGF2(1)
IGF2(2)
IGF2(3)
IGF2(4)
IGF2(5)
IGF2(6)
IGF2(7)
IGF2(8)
IGF2(9)
IGF2(10)
IGF2(11)
IGF2-AS
PRJNA597972
GSE124484
GSE92433
PRJNA721126
GSE93855

B

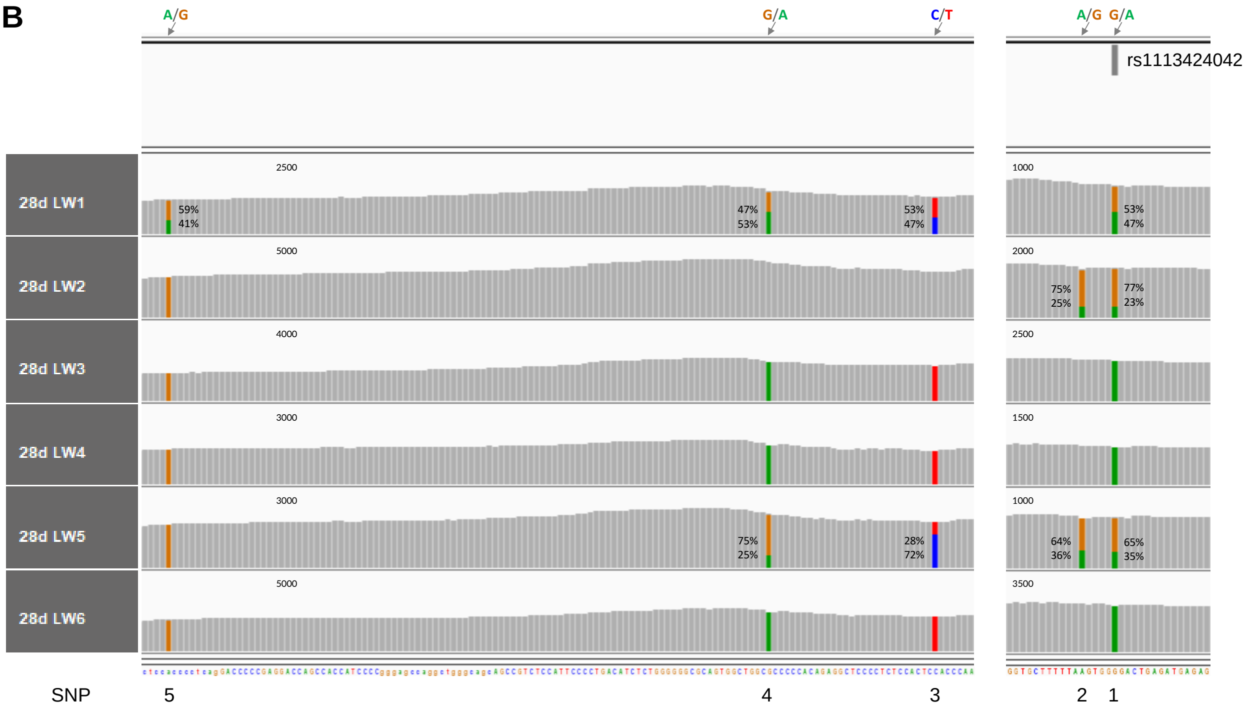

SNP	Genomic coordinate<br>Transcript: Exon	rs ID	Allele		Pig	Liver (mRNA)	
			Ref	Alt		Ref	Alt
1	chr2:1,471,562<br>IGF2(8): E6 / IGF2(3): E4	rs1113424042	G	A	28d LW1	309 (53%)	279 (47%)
					28d LW2	924 (77%)	281 (23%)
					28d LW5	406 (65%)	251 (35%)
2	chr2:1,471,557<br>IGF2(8): E6 / IGF2(3): E4	NR	A	G	28d LW2	299 (25%)	894 (75%)
					28d LW5	220 (36%)	393 (64%)
3	chr2:1,470,430<br>IGF2(8): E6 / IGF2(3): E4	NR	C	T	28d LW1	533 (47%)	612 (53%)
					28d LW5	1206 (72%)	466 (28%)
4	chr2:1,470,402<br>IGF2(8): E6 / IGF2(3): E4	NR	G	A	28d LW1	619 (47%)	690 (53%)
					28d LW5	1484 (75%)	484 (25%)
5	chr2:1,470,301<br>IGF2(8): E6 / IGF2(3): E4	NR	A	G	28d LW1	430 (41%)	615 (59%)

rs, reference SNP; NR, no record; Ref, reference allele; Alt, alternative allele.

Supplementary Figure 4. Changes of expressed *IGF2* mRNA transcripts in the pig liver across various developmental stages. **(A)** The liver tissues are from 1-day-old (1d), 7-day-old (7d), 28-day-old (28d), 70-day-old (70d), 90-day-old (90d), 270-day-old (270d), and 1-year-old (1yr) pigs. Accession numbers of analyzed RNA-seq datasets are listed on the right and data processing procedures are described in Materials and Methods. Red perpendicular arrows within a red bracket indicate sites of biallelic expression analyzed in B. **(B)** Allelic changes in 28d LW pigs. A biallelic tendency in the 28d LW1 pig (due possibly to low monoallelic *IGF2(3)* expression in A and subsequent more contribution of biallelic *IGF2(8)* expression to the allelic ratio) and a decreased biallelic tendency in 28d LW2 and LW5 pigs (due possibly to high monoallelic *IGF2(3)* expression in A and subsequent less contribution of biallelic *IGF2(8)* expression to the allelic ratio) are shown. LW, Large White; LD, Landrace; JH, Jinhua; EB, Enshi black; RC, Rongchang pigs.

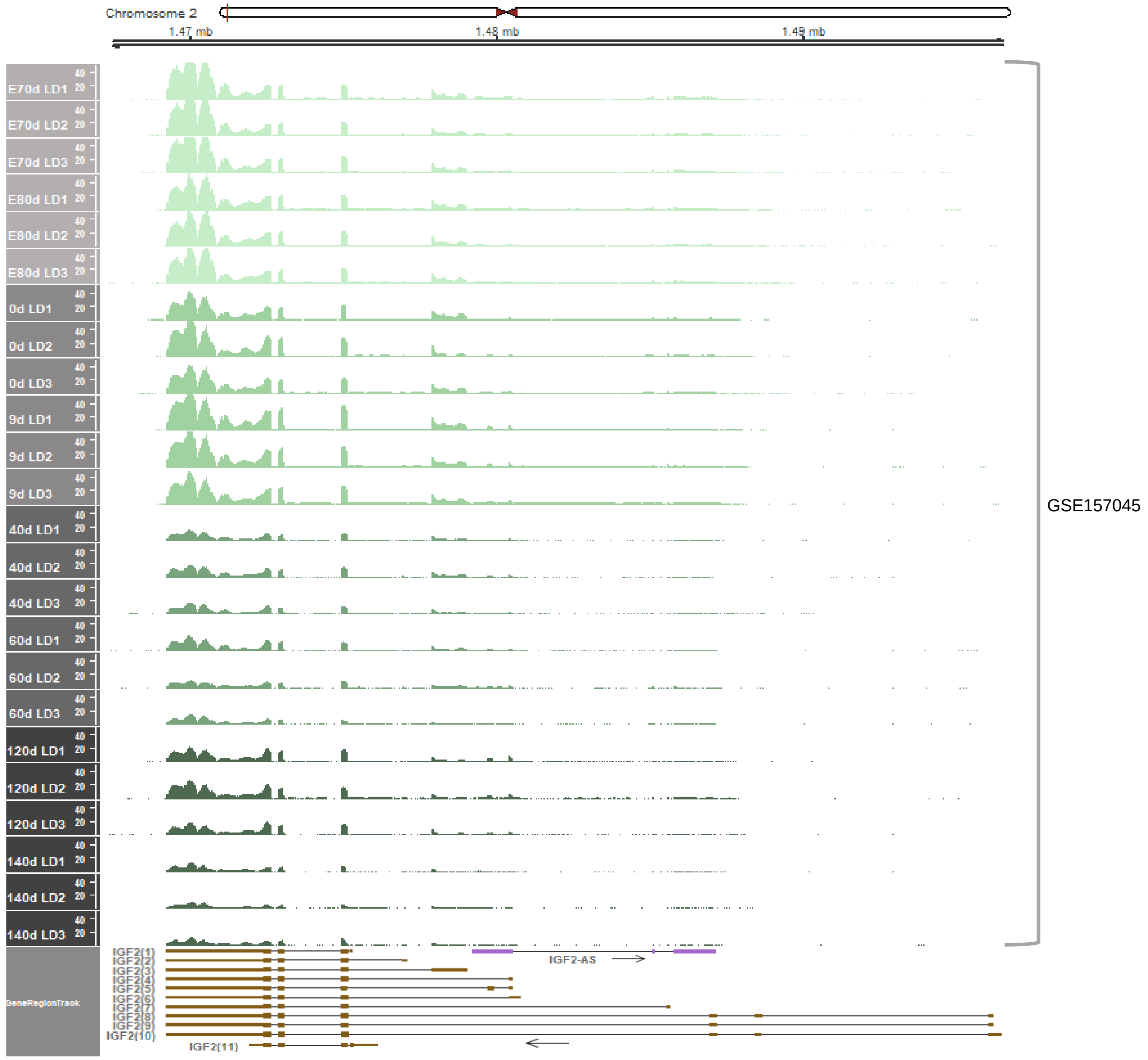

Supplementary Figure 5. Profiling of mRNA expression in the pig skeletal muscle in various developmental stages. The skeletal muscle tissues are from embryonic day 70 (E70d), embryonic day 80 (E80d), 0-day-old (0d), 9-day-old (9d), 40-day-old (40d), 60-day-old (60d), 120-day-old (120d), and 140-day-old (140d) Landrace (LD) pigs. An accession number of the analyzed RNA-seq dataset, which was processed as described in Materials and Methods, is shown on the right.

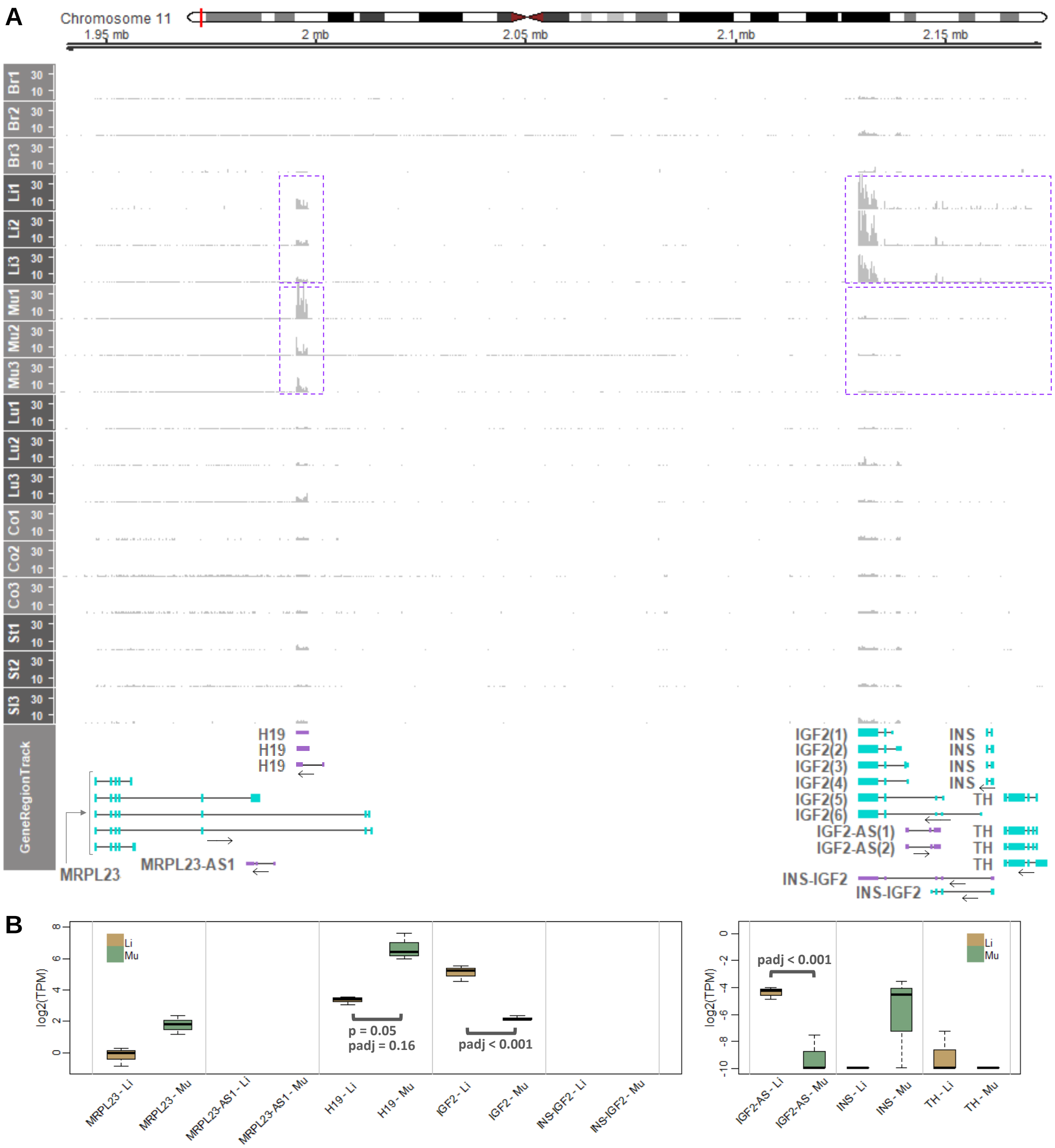

Supplementary Figure 6. Profiling of expression in the *H19/IGF2* locus of adult humans (aged 20-50). **(A)** mRNA expression based on RNA-seq read coverages is represented as TPM (y-axis). Br, brain; Li, liver; Mu, skeletal muscle; Lu, lung; Co, colon; St, stomach. Read coverages around *H19* and *IGF2* in the liver and skeletal muscle are highlighted with purple dotted rectangles. **(B)** Differential expression in between the liver and skeletal muscle with statistical significance (padj less than 0.05). Gene-level quantification on RNA-seq data (GSE120795) was performed before normalization and log2-transformation of TPM values on y-axis. Run IDs of RNA-seq data include: SRR7961214, SRR7961216, and SRR7961218 (brain); SRR7961208, SRR7961209, and SRR7961210 (liver); SRR7961251, SRR7961268, and SRR7961305 (skeletal muscle); SRR7961219, SRR7961220, and SRR7961221 (lung); SRR7961201, SRR7961203, and SRR7961204 (colon); SRR7961238, SRR7961239, and SRR7961247 (stomatch).

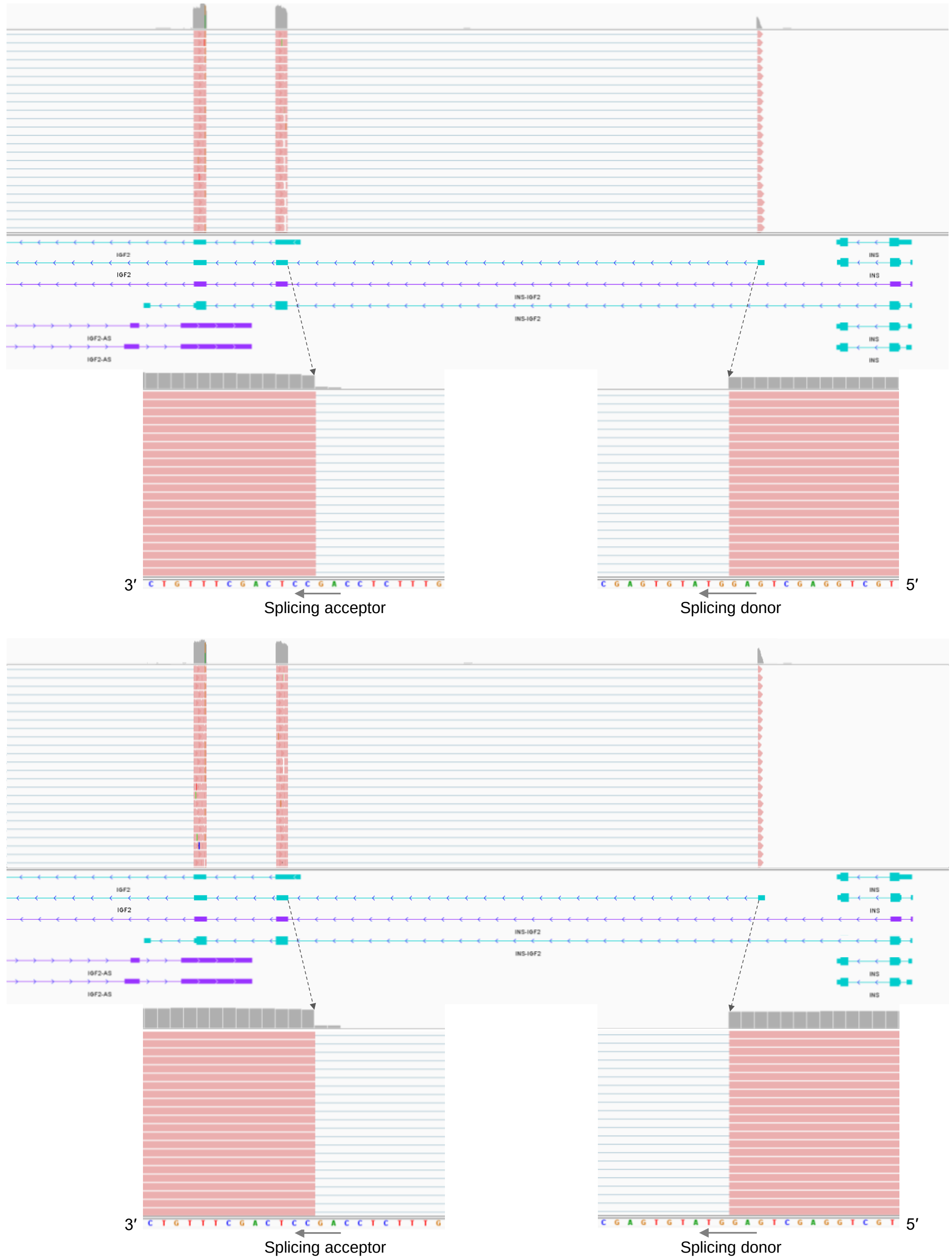

Supplementary Figure 7. Splicing of the human *IGF2* long-form (*IGF2(6)*). The splicing donor (AG/GT) at the end of the 1st exon and splicing acceptor (AG/C) at the beginning of the 2nd exon are shown. Images were obtained from Integrative Genomics Viewer (IGV) and are representatives of nine independent adult normal liver samples.

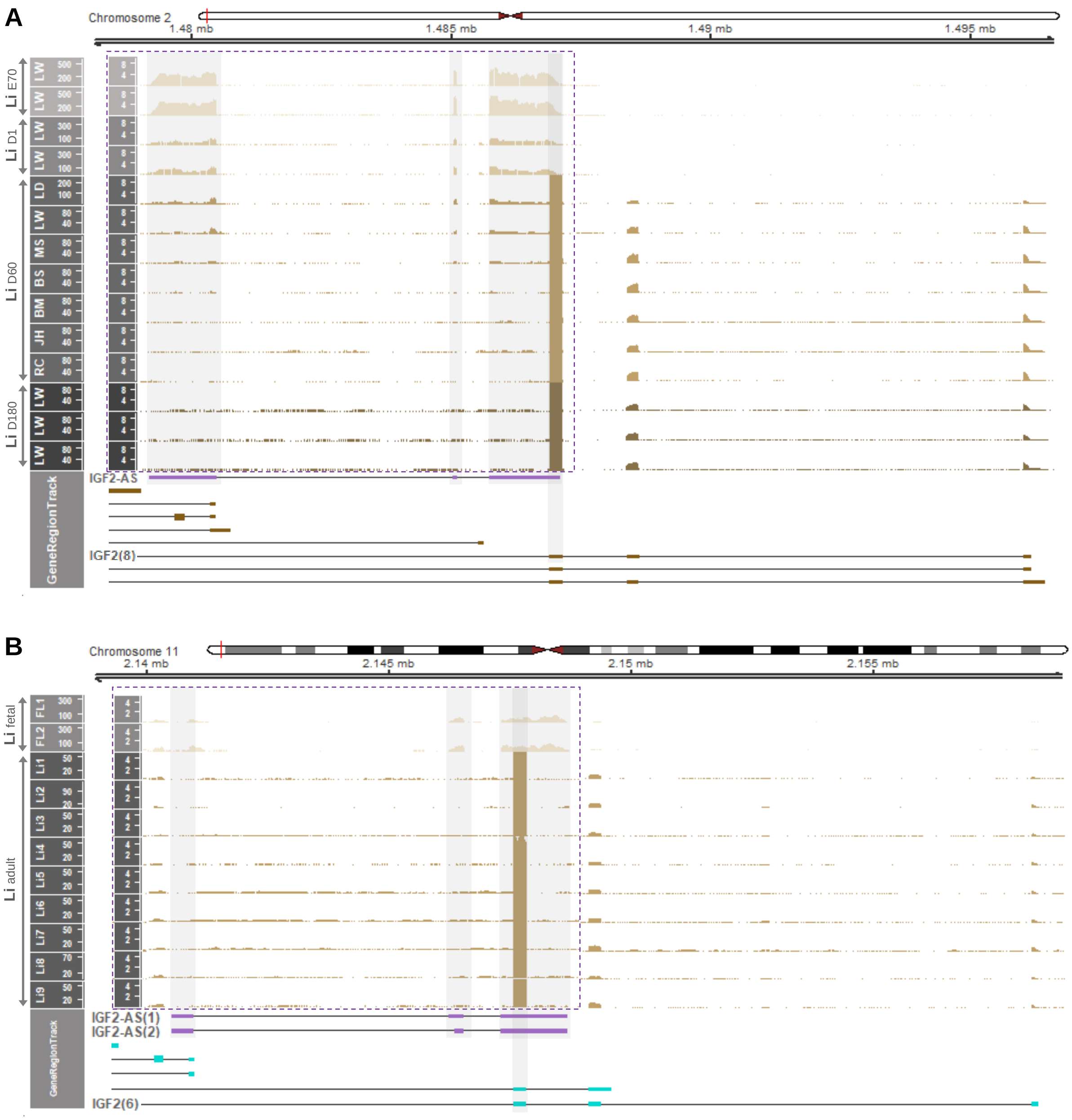

Supplementary Figure 8. Expression of *IGF-AS* in the liver of pigs (A) and humans (B). Additional y-axes inside dotted rectangles represent TPM values for *IGF-AS* transcripts. Read coverages for *IGF2-AS* and overlapping exons of porcine *IGF2(8)* and human *IGF2(6)* are highlighted with grey shades. Details are in the legends of Figs 3 and 5.

A

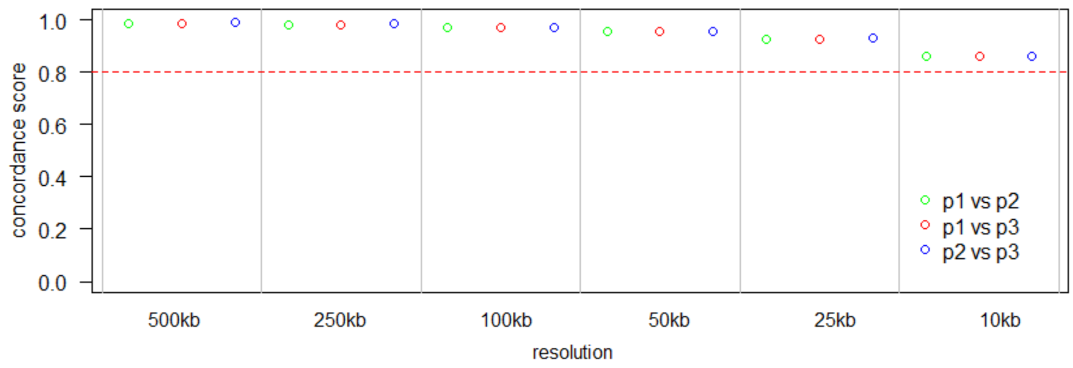

B

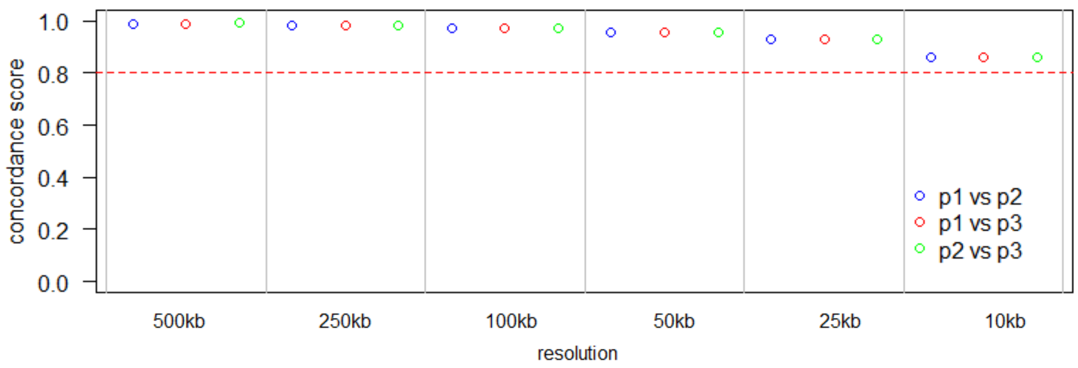

Supplementary Figure 9. Concordance between Hi-C contact maps. Hi-C data of three replicates of the fetal (A) and adult (B) livers from Bamaxiang pigs (p1-3 each) were retrieved under PRJNA482496 and assessed using GenomeDISCO. Raw matrices at six resolutions (500 kb, 250 kb, 100 kb, 50 kb, 25 kb, and 10 kb) were compared in a pairwise manner. The concordance threshold score (0.8) is indicated with red dotted lines.

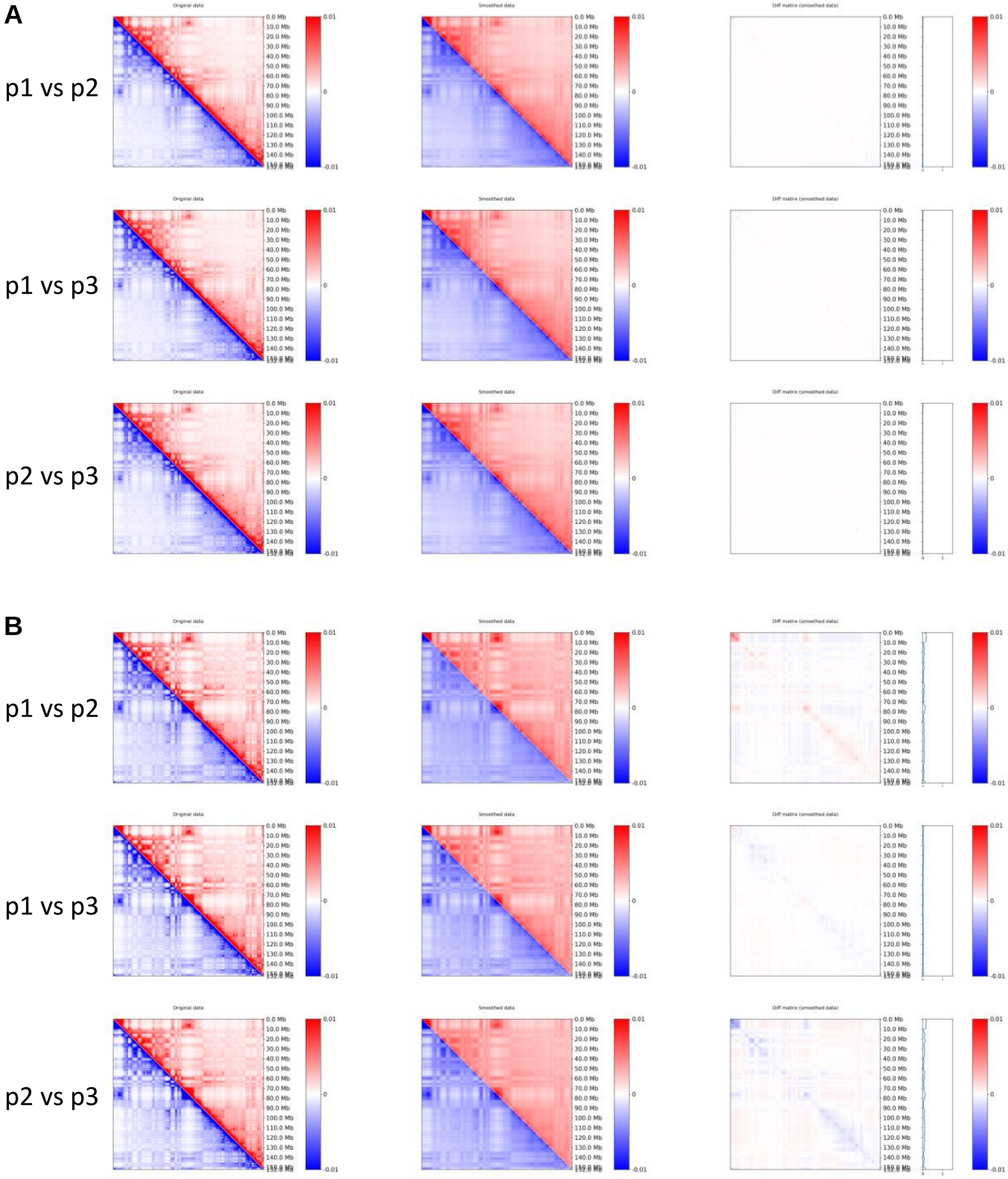

Supplementary Figure 10. Pairwise comparisons of Hi-C matrices of pig chromosome 2 at a 500-kb resolution. GenomeDISCO was used to compare liver tissues from three fetal **(A)** and adult **(B)** Bamaxiang pigs (p1-3 each). Original matrices (left) were randomly walked to generate smoothed contact maps (middle) to estimate differential matrices and concordance of the data (right).

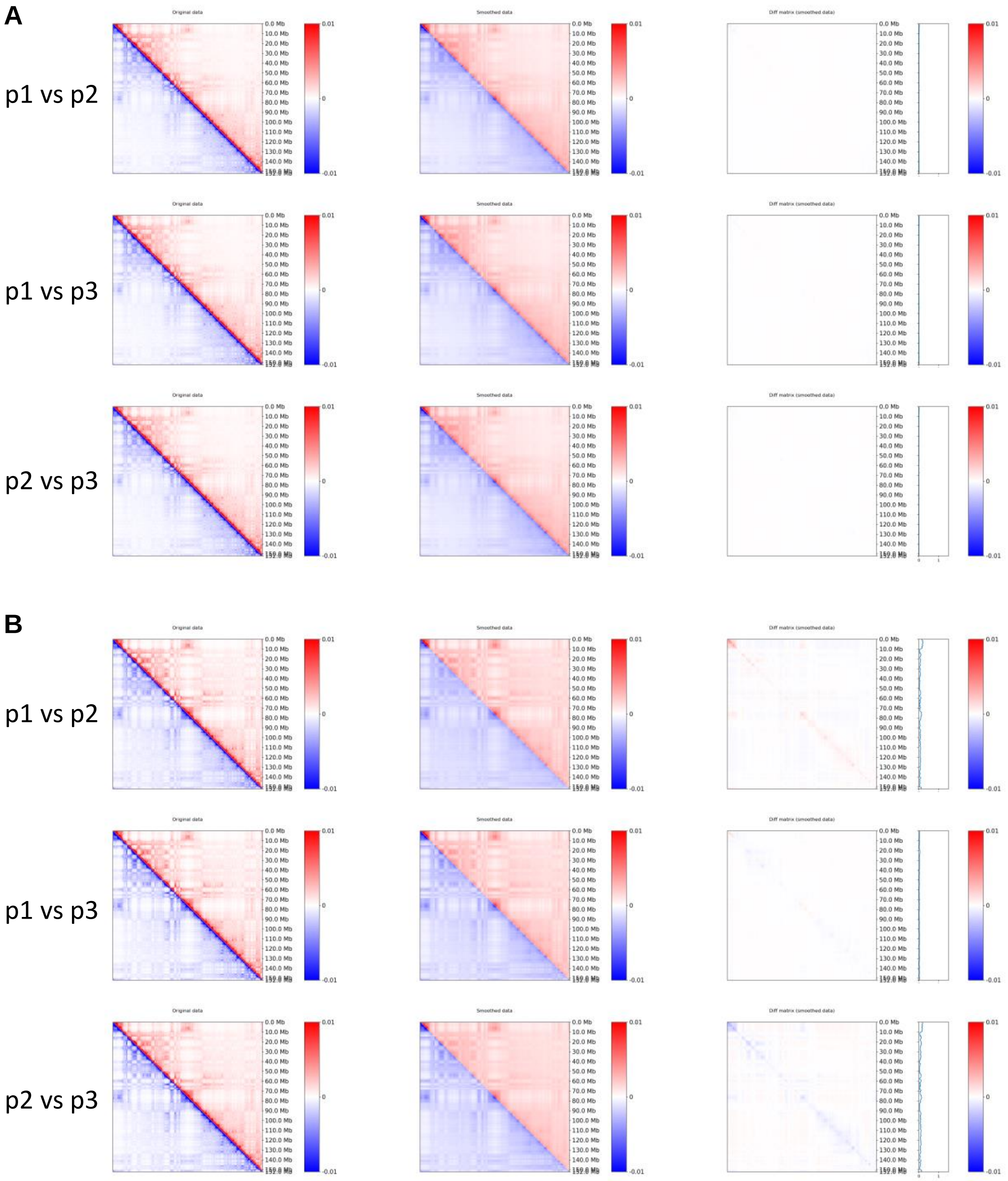

Supplementary Figure 11. Pairwise comparisons of Hi-C matrices of pig chromosome 2 at a 250-kb resolution. GenomeDISCO was used to compare liver tissues from three fetal **(A)** and adult **(B)** Bamaxiang pigs (p1-3 each). Original matrices (left) were randomly walked to generate smoothed contact maps (middle) to estimate differential matrices and concordance of the data (right).

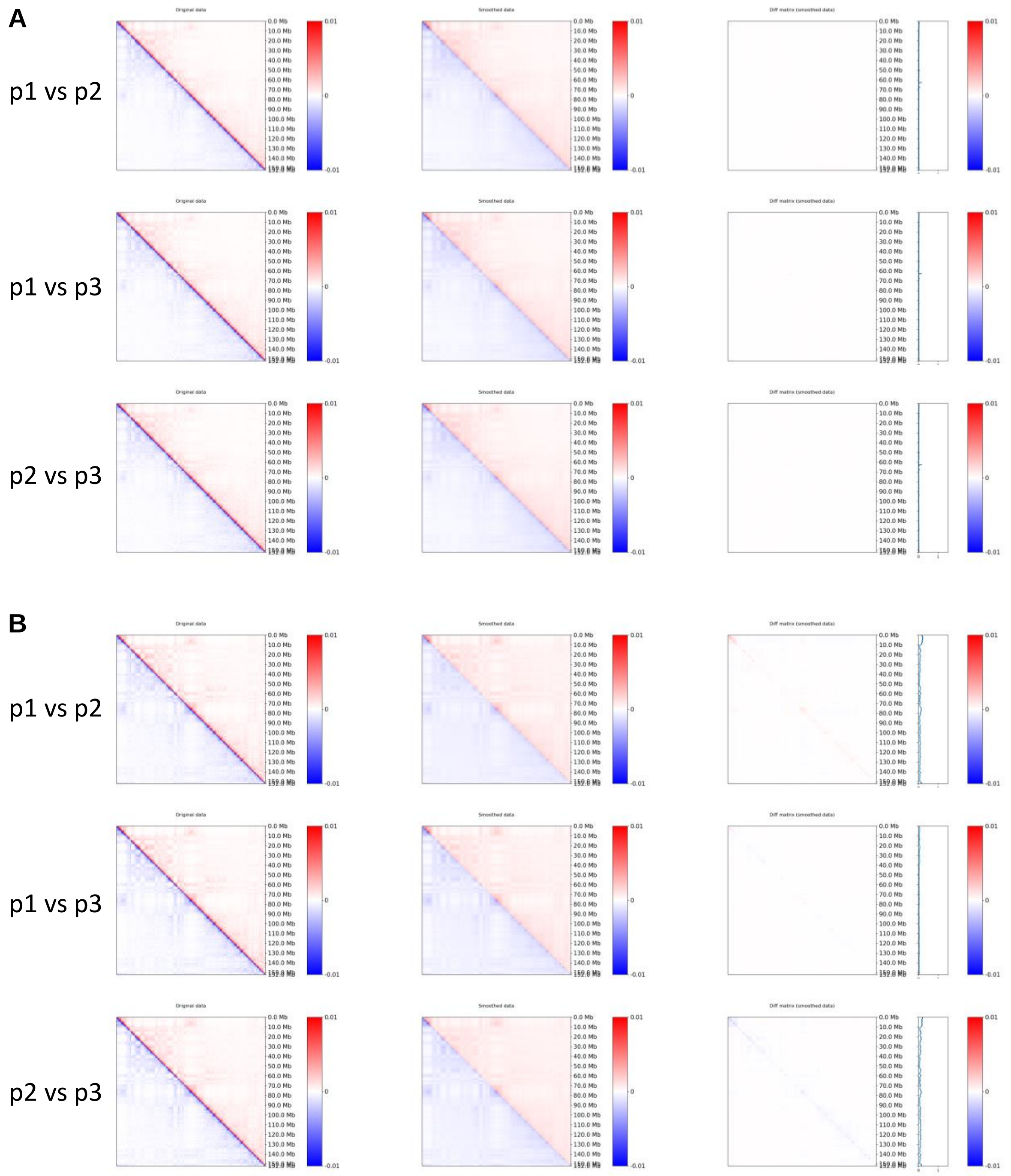

Supplementary Figure 12. Pairwise comparisons of Hi-C matrices of pig chromosome 2 at a 100-kb resolution. GenomeDISCO was used to compare liver tissues from three fetal **(A)** and adult **(B)** Bamaxiang pigs (p1-3 each). Original matrices (left) were randomly walked to generate smoothed contact maps (middle) to estimate differential matrices and concordance of the data (right).

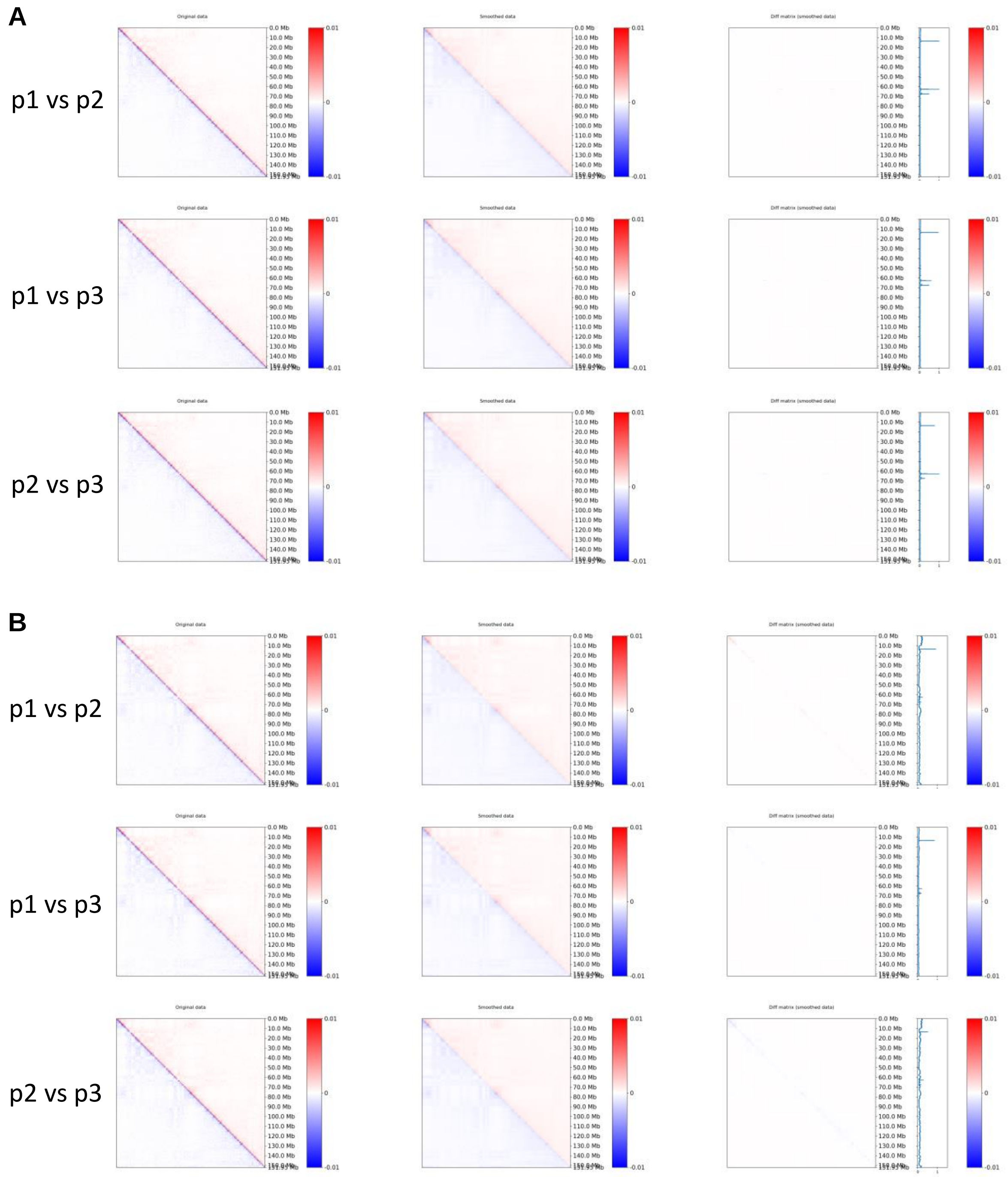

Supplementary Figure 13. Pairwise comparisons of Hi-C matrices of pig chromosome 2 at a 50-kb resolution. GenomeDISCO was used to compare liver tissues from three fetal **(A)** and adult **(B)** Bamaxiang pigs (p1-3 each). Original matrices (left) were randomly walked to generate smoothed contact maps (middle) to estimate differential matrices and concordance of the data (right).

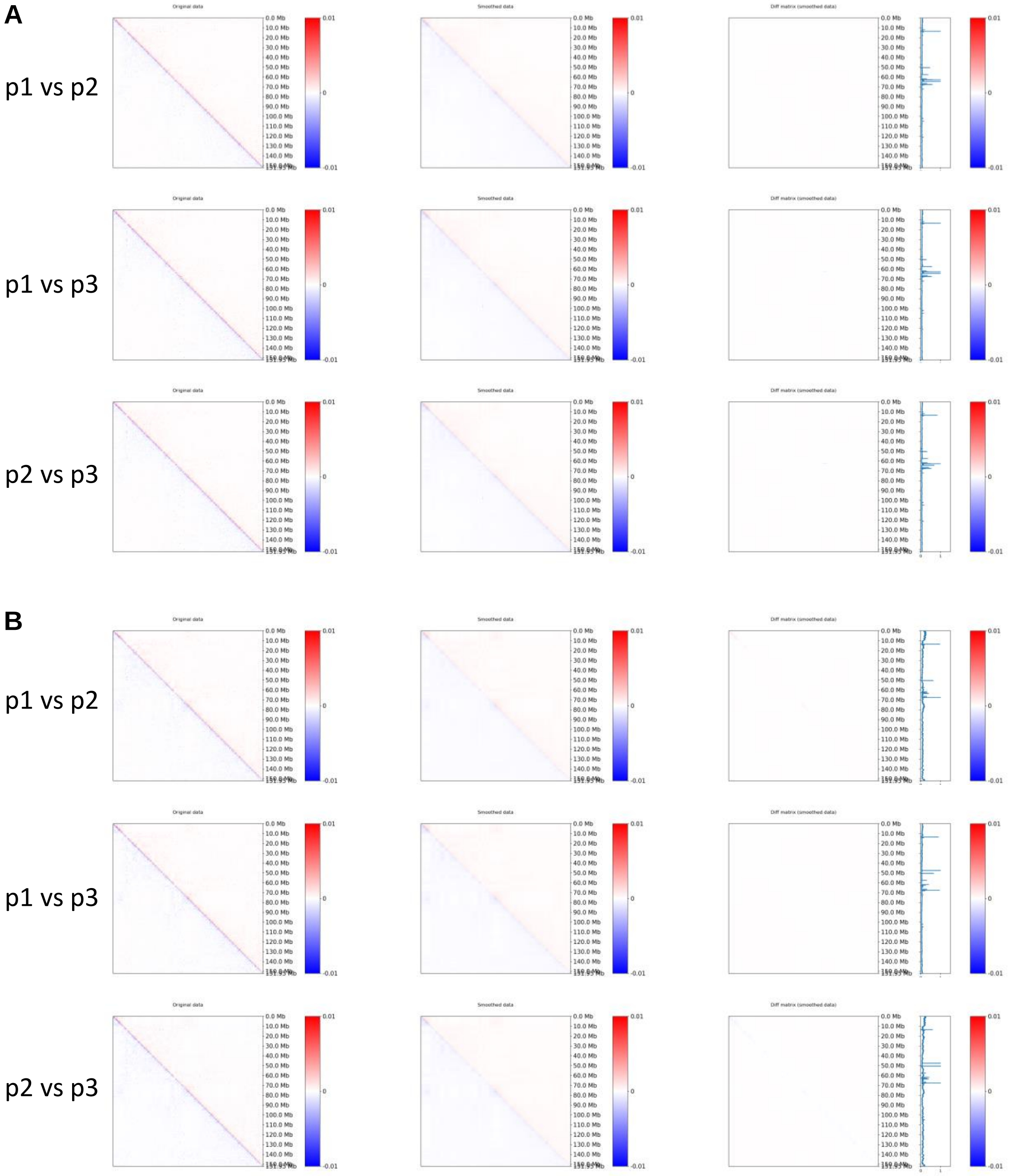

Supplementary Figure 14. Pairwise comparisons of Hi-C matrices of pig chromosome 2 at a 25-kb resolution. GenomeDISCO was used to compare liver tissues from three fetal **(A)** and adult **(B)** Bamaxiang pigs (p1-3 each). Original matrices (left) were randomly walked to generate smoothed contact maps (middle) to estimate differential matrices and concordance of the data (right).

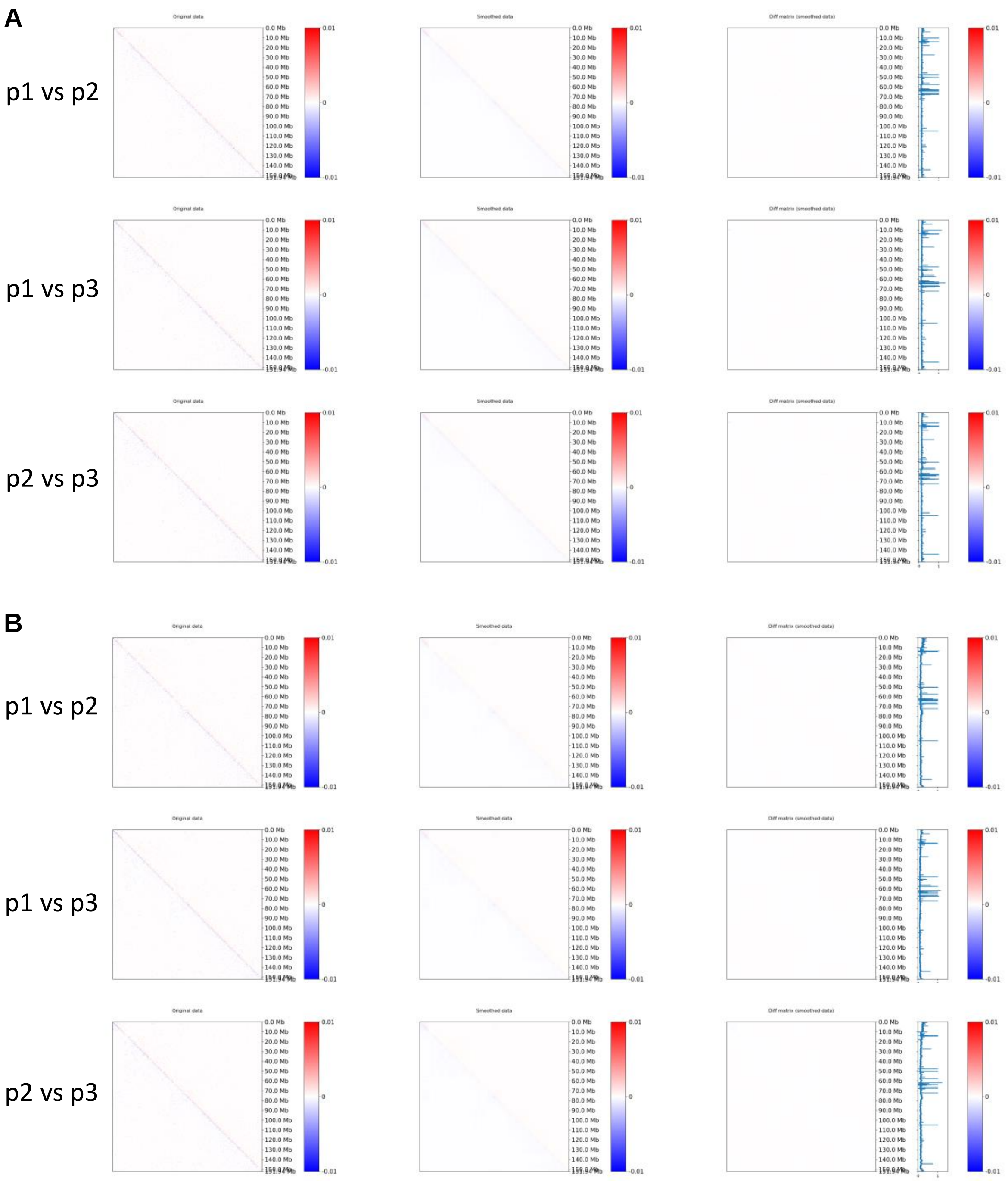

Supplementary Figure 15. Pairwise comparisons of Hi-C matrices of pig chromosome 2 at a 10-kb resolution. GenomeDISCO was used to compare liver tissues from three fetal **(A)** and adult **(B)** Bamaxiang pigs (p1-3 each). Original matrices (left) were randomly walked to generate smoothed contact maps (middle) to estimate differential matrices and concordance of the data (right).

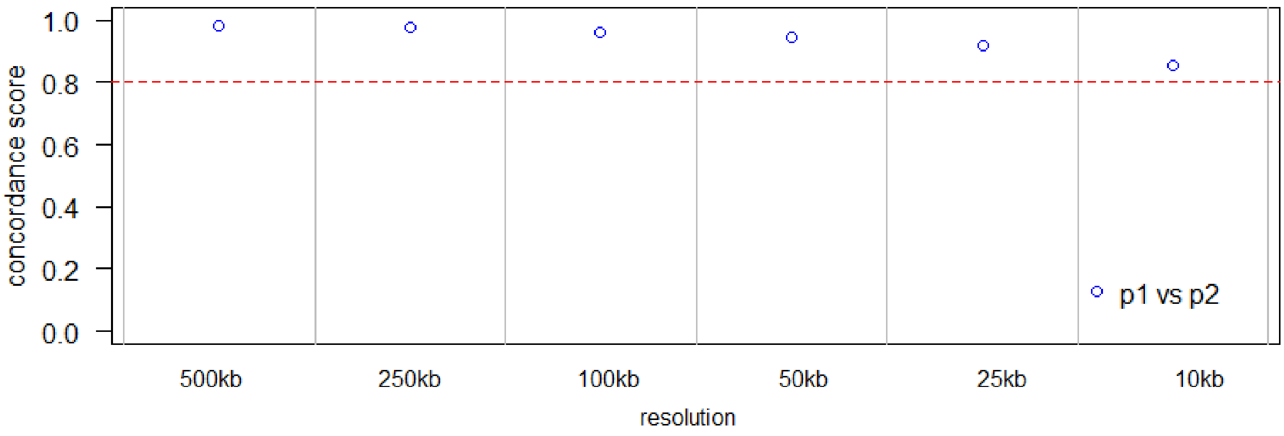

Supplementary Figure 16. Concordance between Hi-C contact maps. Hi-C data of two replicates of skeletal muscle from two 2-weeks-old Large White pigs (p1 and p2) were retrieved under GSE143288. Raw matrices at six resolutions (500 kb, 250 kb, 100 kb, 50 kb, 25 kb, and 10 kb) were compared within each pair using GenomeDISCO. A red dotted line marks the concordance threshold (0.8).

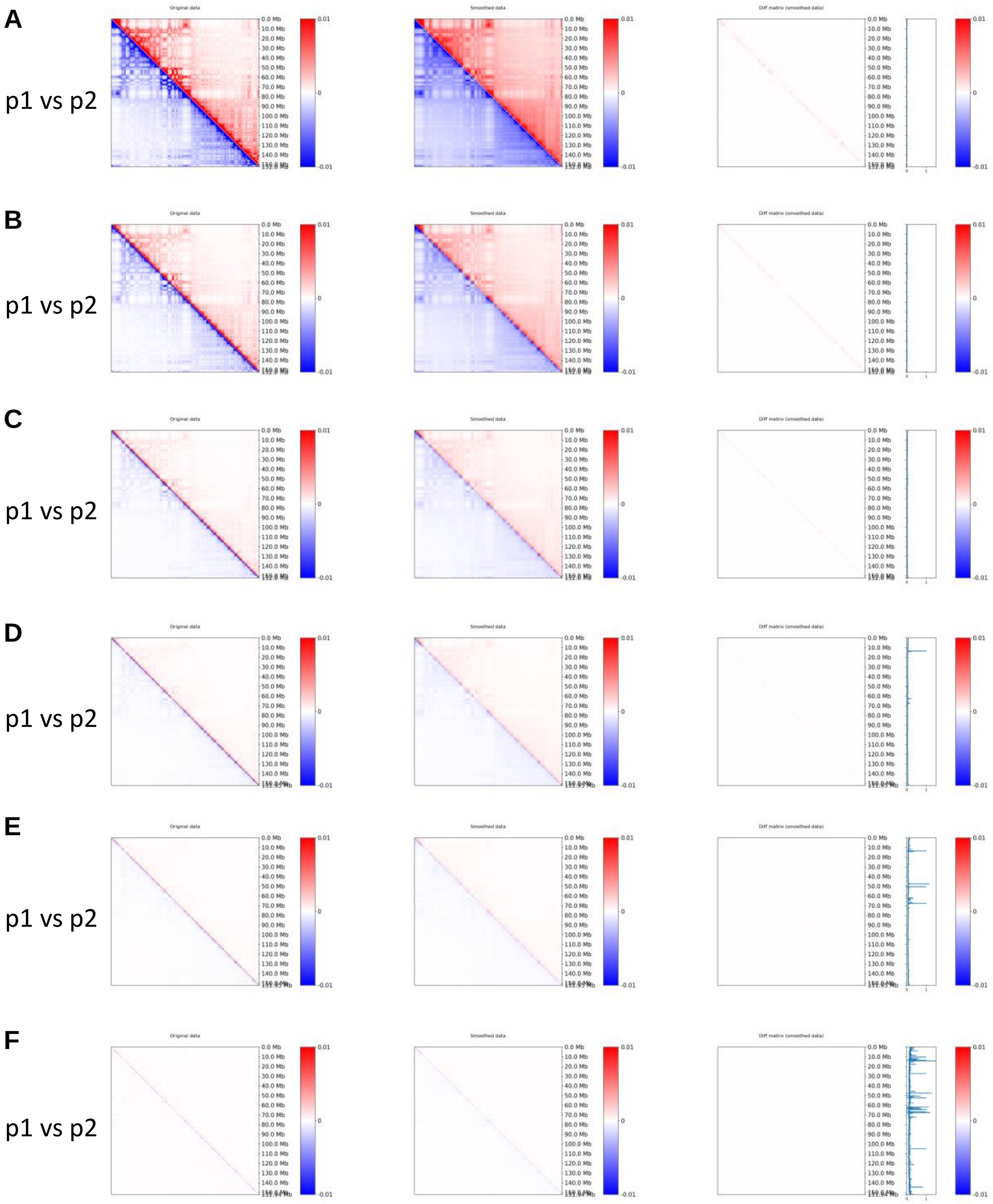

Supplementary Figure 17. Pairwise comparisons of Hi-C matrices of pig chromosome 2 at 500-kb **(A)**, 250-kb **(B)**, 100-kb **(C)**, 50-kb **(D)**, 25-kb **(E)**, and 10-kb **(F)** resolutions. GenomeDISCO was used to compare matrices of skeletal muscle tissues from two 2-weeks-old Large White pigs (p1 and p2). Original matrices (left) were randomly walked to generate smoothed contact maps (middle) to estimate differential matrices and concordance of the data (right).

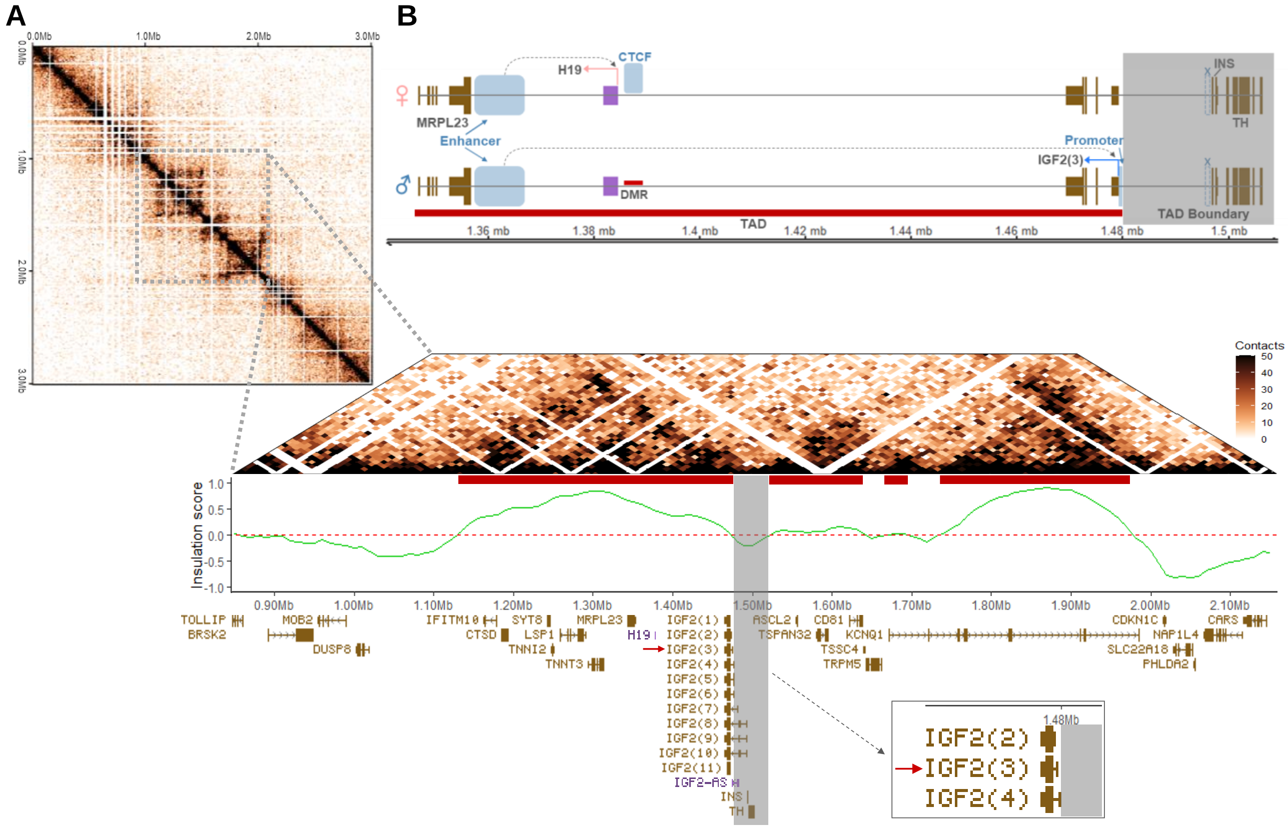

Supplementary Figure 18. Chromatin interaction and TADs in the muscle of fetal pigs. **(A)** At a 10-kb resolution, the ICE normalized Hi-C matrix of fetal muscle are displayed. A dataset under GSE166346 was retrieved. Without merging, one deeply sequenced matrix was processed for the fetal muscle. This matrix was zoomed in using a truncated pyramid plot. TADs identified using insulation scores are represented with red bars, and a TAD boundary in the *IGF2* locus is indicated with the grey perpendicular shades. A red arrow denotes the predominantly expressed transcript, *IGF2(3)*, in the muscle of fetal pigs. **(B)** Schematic representation of the *H19/IGF2* locus of porcine fetal muscle displaying long-range gene regulation and chromatin interaction.

1.2 Supplementary Tables

Supplementary Table 1. Summary of WGBS and RNA-seq data

WGBS summary

Sequencing reads				Bisulfite conversion rate			Paired-end alignment	
Sample	Total reads	Trimmed reads	GC (%)	# unmethylated read level measurements from lambda phage genome	# methylated read level measurements from lambda phage genome	Estimated bisulfite conversion rate (%)	Uniquely mapped reads (% out of trimmed reads)	Deduplicated reads (% out of uniquely mapped reads)
PA1	841,577,694	839,707,296	22.00	52,500,445	150,809	99.71	680,816,512 (81.08 %) (340,408,256 in pairs)	579,931,666 (85.18 %) (289,965,833 in pairs)
PA2	858,565,284	856,896,438	22.00	79,675,768	233,291	99.71	690,861,896 (80.62 %) (345,430,948 in pairs)	602.112,770 (87.15 %) (301,056,385 in pairs)
PA3	851,137,894	849,155,508	22.00	54,455,017	157,706	99.71	676,257,914 (79.64 %) (338,128,957 in pairs)	588,335,740 (87.00 %) (294,167,870 in pairs)
CN1	848,497,920	846,508,748	21.50	74,074,285	232,693	99.69	666,991,828 (78.79 %) (333,495,914 in pairs)	571,615,706 (85.70 %) (285,807,853 in pairs)
CN2	863,763,714	862,085,926	22.00	52,790,995	152,946	99.71	700,686,714 (81.28 %) (350,343,357 in pairs)	594,127,426 (84.79 %) (297,063,713 in pairs)
CN3	868,440,532	866,479,482	22.00	51,678,880	152,823	99.71	694577450 (80.16 %) (347,288,725 in pairs)	593273066 (85.41 %) (296,636,533 in pairs)

GC (%): GC Content

unmethylated read level measurements from lambda phage genome : converted from Cs to Ts

methylated read level measurements from lambda phage genome: unconverted methylated Cs

Estimated bisulfite conversion rate (%): converted/(converted + unconverted)x 100 = Ts/(Ts + Cs)x100

RNA-seq summary

Sequencing reads				Paired-end alignment
Sample	Total reads	Trimmed reads	GC (%)	Filtered reads (Deduplicated & MAPQ > 30) (% out of trimmed reads)
PA1	80,726,836	80,467,810	51.00	58,616,608 (72.84 %) (29,308,304 in pairs)
PA2	79,851,570	79,630,362	51.00	59,050,813 (74.16 %) (29,525,406 in pairs)
PA3	80,970,502	80,731,616	51.00	61,963,122 (76.75 %) (30,981,561 in pairs)
CN1	77,314,870	77,147,412	50.00	57,086,163 (74.00 %) (28,543,081 in pairs)
CN2	73,465,582	73,314,974	51.00	57,086,399 (77.86 %) (28,543,199 in pairs)
CN3	77,689,426	77,547,380	51.00	59,513,132 (76.74 %) (29,756,566 in pairs)

GC (%): GC Content

Supplementary Table 2. DMRs calling using metilene

Methylated regions and DMRs adjacent to and within the *H19/IGF2* locus. Red highlights are on DMRs (q-value < 0.05).

chr	start	end	q-value	mean methyl difference	#CpGs	p-value (MWU)	p-value (2D KS)	mean g1	mean g2
chr2	1353031	1353239	1	0.130678	15	0.042404	0.030133	0.86336	0.73268
chr2	1354107	1354215	1	-0.175794	10	0.078519	0.074211	0.74056	0.91635
chr2	1354296	1354591	1	0.194511	12	0.028897	0.081683	0.79405	0.59954
chr2	1355078	1355162	1	0.146818	16	0.046249	0.085177	0.7652	0.61838
chr2	1357110	1357205	1	0.130507	14	0.015334	0.036562	0.89382	0.76331
chr2	1359300	1359385	1	0.124095	12	0.012411	0.062044	0.76768	0.64358
chr2	1359509	1359601	1	0.114602	11	0.071571	0.172	0.53213	0.41753
chr2	1359873	1360124	0.60048	0.276768	11	0.00083545	0.0030646	0.79596	0.51919
chr2	1360211	1360252	1	0.250516	10	0.0026888	0.053614	0.79944	0.54893
chr2	1361464	1361566	1	-0.102447	12	0.17297	0.41782	0.51445	0.6169
chr2	1361612	1361698	1	0.103333	10	0.74499	0.65308	0.825	0.72167
chr2	1362063	1362143	1	0.168056	10	0.01247	0.10074	0.74016	0.5721
chr2	1362187	1362343	0.93527	0.243948	20	8.64E-05	0.0091603	0.85986	0.61591
chr2	1362681	1362795	1	0.126424	11	0.013082	0.030844	0.8035	0.67708
chr2	1365278	1365355	1	0.104577	10	0.024625	0.10076	0.99333	0.88876
chr2	1368483	1368556	1	0.100652	13	0.086554	0.21946	0.6067	0.50605
chr2	1368579	1368711	0.42044	0.184229	13	0.0004964	0.0014675	0.82373	0.6395
chr2	1369004	1369063	1	0.14746	10	0.022798	0.05368	0.87409	0.72663
chr2	1369090	1369240	1	0.136089	12	0.056998	0.081239	0.78823	0.65214
chr2	1369590	1369971	0.98061	0.184976	28	0.00063428	0.0096728	0.56325	0.37828
chr2	1370272	1370408	1	0.138016	10	0.066763	0.13316	0.80222	0.66421
chr2	1370408	1370568	0.26802	0.265537	14	6.13E-05	0.00059104	0.81417	0.54864
chr2	1370608	1370800	1	-0.227579	12	0.0092793	0.0614	0.11204	0.33962
chr2	1370934	1371130	1	0.138611	10	0.089092	0.093828	0.69556	0.55694
chr2	1372441	1372660	0.90785	0.218059	10	0.0019045	0.0086341	0.81897	0.60091
chr2	1373091	1373163	1	0.156548	10	0.018737	0.053565	0.80552	0.64897
chr2	1373850	1373967	1	0.114141	10	0.06899	0.097098	0.84371	0.72956
chr2	1375392	1375507	1	-0.109603	10	0.019494	0.012584	0.12968	0.23929
chr2	1376124	1376221	1	-0.139116	14	0.1093	0.17368	0.15539	0.2945
chr2	1376229	1376289	0.90785	-0.259921	12	0.0012281	0.0087287	0.15443	0.41435
chr2	1376709	1376770	1	0.237169	10	0.00041013	0.018734	0.92122	0.68405
chr2	1378322	1378374	0.67954	0.217274	17	0.0012706	0.0042441	0.87941	0.66214
chr2	1378416	1378510	0.66828	0.246712	14	0.0013198	0.0041251	0.79484	0.54813
chr2	1378537	1378602	0.22993	0.389286	14	1.36E-06	0.00041421	0.78452	0.39524
chr2	1378632	1378948	1	0.171967	15	0.017476	0.066919	0.86995	0.69798
chr2	1382916	1383071	1	0.110654	11	0.33292	0.28916	0.90055	0.7899
chr2	1384525	1385289	1.76E-36	-0.365887	102	5.13E-14	1.31E-41	0.12346	0.48935
chr2	1385381	1385787	8.34E-29	-0.469294	47	4.97E-14	8.05E-34	0.030851	0.50014
chr2	1385811	1385919	0.01861	-0.298161	10	1.81E-05	6.28E-06	0.072341	0.3705
chr2	1385929	1387139	2.19E-142	-0.461314	185	5.03E-14	8.86E-135	0.039949	0.50126
chr2	1387139	1387385	9.21E-05	-0.359826	15	8.48E-12	4.93E-09	0.13313	0.49296
chr2	1387471	1387716	5.24E-08	-0.439008	18	4.13E-14	8.56E-13	0.075533	0.51454
chr2	1388807	1388894	0.6335	0.143179	13	0.0017906	0.0034203	0.85073	0.70755
chr2	1390551	1390811	1	0.109535	12	0.037204	0.17918	0.89452	0.78499
chr2	1393394	1393537	1	0.11627	10	0.027603	0.099509	0.87405	0.75778
chr2	1394360	1394419	1	0.128558	10	0.035783	0.018602	0.82187	0.69331
chr2	1394652	1394759	1	0.128282	12	0.019152	0.015901	0.71835	0.59007
chr2	1394853	1394961	1	0.129905	17	0.012666	0.051981	0.78695	0.65704
chr2	1396493	1396692	1	0.127685	12	0.078932	0.17874	0.39048	0.26279
chr2	1399265	1399628	0.00028721	0.240178	52	1.28E-13	1.84E-08	0.8908	0.65062
chr2	1399636	1399743	1	0.108637	12	0.052731	0.42281	0.83069	0.72205
chr2	1400445	1400659	0.90785	0.158827	10	0.0056977	0.0086201	0.95565	0.79683
chr2	1402780	1402984	1	0.174931	12	0.0050424	0.10784	0.84446	0.66952
chr2	1406760	1406856	1	0.142011	10	0.015323	0.073934	0.9496	0.80759
chr2	1412922	1413025	1	0.120577	10	0.019494	0.072819	0.88132	0.76074
chr2	1414158	1414284	0.90785	0.151414	12	0.15919	0.0087856	0.79706	0.64565
chr2	1419085	1419136	1	0.110708	13	0.0043294	0.048411	0.91313	0.80242
chr2	1428579	1428784	0.12615	0.179092	15	3.30E-05	0.0001491	0.83841	0.65932
chr2	1429067	1429230	1	-0.128439	10	0.29386	0.45695	0.70278	0.83122
chr2	1430637	1430775	1	0.126241	10	0.030887	0.1744	0.87579	0.74955
chr2	1431711	1431863	1	0.105246	11	0.015631	0.1039	0.91965	0.8144
chr2	1432672	1432980	0.79739	-0.152337	18	0.00066399	0.0062771	0.77017	0.92251
chr2	1435698	1435803	1	0.140873	10	0.0032599	0.012823	0.89967	0.7588
chr2	1436044	1436141	1	0.162696	10	0.0062358	0.053142	0.95547	0.79278
chr2	1439064	1439163	0.10979	-0.336367	10	1.07E-06	0.00011342	0.38498	0.72135
chr2	1440059	1440359	1	0.128986	15	0.036977	0.085509	0.89343	0.76444
chr2	1441050	1441100	1	0.175426	12	0.0021888	0.032593	0.6844	0.50898
chr2	1443613	1443784	1	0.138733	11	0.034891	0.13786	0.86655	0.72781
chr2	1444639	1444752	1	0.121935	12	0.14627	0.42162	0.82233	0.7004
chr2	1445008	1445354	1	0.109432	12	0.0047011	0.13986	0.83535	0.72592
chr2	1450627	1450761	1	-0.160362	12	0.0081296	0.024436	0.26103	0.4214
chr2	1450896	1450972	1	-0.153862	10	0.020278	0.072863	0.19296	0.34683
chr2	1451154	1451326	1	-0.140952	10	0.13929	0.36889	0.55111	0.69206
chr2	1452812	1453381	1	-0.164104	16	0.010548	0.067156	0.61283	0.77693
chr2	1455297	1455457	1	0.149482	12	0.012022	0.061544	0.75815	0.60867
chr2	1463623	1463801	1	0.179996	10	0.01247	0.053744	0.30437	0.12437
chr2	1467381	1467660	1	0.134015	13	0.028995	0.17697	0.88974	0.75573
chr2	1468254	1468459	0.7702	0.150904	12	0.0035349	0.0059519	0.82207	0.67116
chr2	1468471	1468566	1	0.153836	13	0.015376	0.026665	0.9098	0.75596
chr2	1471786	1472030	1	0.166546	10	0.010993	0.10019	0.88959	0.72305
chr2	1472561	1472688	1	0.12	10	0.047579	0.052771	0.74889	0.62889
chr2	1474788	1474867	1	0.159259	10	0.011467	0.038334	0.9154	0.75614
chr2	1475126	1475215	1	0.172859	10	0.0014796	0.074024	0.80156	0.62871
chr2	1475254	1475308	0.50218	0.156018	10	0.00021893	0.0024063	0.88353	0.72751
chr2	1475402	1475492	1	0.137619	10	0.0096739	0.052374	0.80204	0.66442
chr2	1475669	1475843	1	-0.120225	10	0.0081351	0.026992	0.78493	0.90516
chr2	1476465	1476594	1	0.164802	10	0.060433	0.13401	0.46845	0.30365
chr2	1485204	1485559	0.50218	0.163795	17	0.00013813	0.0022408	0.87334	0.70955
chr2	1486297	1486379	0.75476	0.135848	10	0.0015569	0.005661	0.91829	0.78245
chr2	1490427	1490502	1	0.16751	10	0.041325	0.096794	0.95496	0.78745

Supplementary Table 3. Differentially expressed genes between PA and CN embryos within the *H19*/*IGF2* locus

	TPM					
Gene	PA ave ± SEM[a]	CN ave ± SEM	PA ave / CN ave	CN ave / PA ave	p-value	p-adj[b]
MRPL23	92.33 ± 4.19	108.91 ± 0.53	0.85	1.18	0.14	0.46
LOC110259183	0.02 ± 0.02	0.02 ± 0.01	1.02	0.98	0.96	NA
H19	8687.28 ± 1796.40	5401.25 ± 438.38	1.61	0.62	0.05	0.24
LOC110259218	0.00 ± 0.00	0.01 ± 0.01	0.00	NA	0.80	NA
LOC110259219	0.00 ± 0.00	0.00 ± 0.00	NA	NA	NA	NA
IGF2	34.36 ± 6.86	1517.48 ± 148.78	0.02	44.16	< 0.001	< 0.001[c]
IGF2-AS	1.37 ± 0.30	14.56 ± 0.96	0.09	10.63	< 0.001	< 0.001
INS	5.72 ± 1.96	6.26 ± 1.92	0.91	1.09	0.80	0.94
TH	4.77 ± 0.48	3.29 ± 0 .24	1.45	0.69	0.02	0.11

[a]Average (ave) ± standard error of the mean (SEM) of TPM in either PA or CN embryos. [b]Benjamini–Hochberg (BH) adjusted *p*-value. [c]Value of less than 0.05 represents statistical significance. NA, not available.

Supplementary Table 4. Read counts of informative SNPs within the *IGF2* transcripts of the pig

SNP	Genomic coordinate & Transcript: Exon	rs ID	Allele		Pig breed	gDNA		He	Liver (mRNA)		S. muscle (mRNA)	
			Ref	Alt		Ref	Alt		Ref	Alt	Ref	Alt
1	chr2:1,496,056<br>IGF2(8): E1	NR	T	C	BM	5 (42%)	7 (58%)	*	420 (45%)	507 (55%)	-	-
2	chr2:1,488,383<br>IGF2(8): E2	rs1113378991	C	T	BM	10 (56%)	8 (44%)	*	186 (41%)	269 (59%)	-	-
3	chr2:1,487,114<br>IGF2(8): E3	NR	C	G	BM	16 (84%)	3 (16%)	*	272 (52%)	252 (48%)	-	-
4	chr2:1,487,011<br>IGF2(8): E3	NR	C	T	LD	6 (75%)	2 (25%)	*	212 (43%)	279 (57%)	-	-
5	chr2:1,486,746	NR	C	T	MS	11 (58%)	8 (42%)	*	0	6 (100%)	0	4 (100%)
	IGF2-AS: E3				RC	10 (53%)	9 (47%)	*	1 (100%)	0	0	4 (100%)
6	chr2:1,472,974<br>IGF2(8): E5<br>IGF2(3): E3	NR	G	A	RC	9 (53%)	8 (47%)	*	636 (55%)	514 (45%)	63 (5%)	1188 (95%)
7	chr2:1,472,384<br>IGF2(8): E6<br>IGF2(3): E4	rs1109870997	G	A	RC	6 (32%)	13 (68%)	*	833 (60%)	551 (40%)	86 (6%)	1451 (94%)
8	chr2:1,472,350<br>IGF2(8): E6<br>IGF2(3): E4	NR	T	A	JH	9 (60%)	6 (40%)	*	472 (55%)	380 (44%)	1535 (90%)	156 (9%)
9	chr2:1,471,557	NR	A	G	LD	6 (67%)	3 (33 %)	*	1271 (84%)	242 (16%)	1187 (99%)	14 (1%)
	IGF2(8): E6				MS	14 (54%)	12 (46%)	*	252 (34%)	497 (66%)	45 (12%)	317 (88%)
	IGF2(3): E4				BM	9 (69%)	4 (31%)	*	235 (50%)	231 (50%)	244 (90%)	27 (10%)

NR, no record; He, heterozygous SNP

Supplementary Table 5. Read counts of informative SNPs within the IGF2 transcripts of the human liver

SNP	Genomic coordinate & Transcript: Exon	rs ID	Allele		Tissue	gDNA		He	Fetal liver (mRNA)		Liver (mRNA)	
			Ref	Alt		Ref	Alt		Ref	Alt	Ref	Alt
1	chr11:2,146,388	rs1003484	A	G	FL1	5 (56%)	4 (44%)	*	0	6 (100%)	-	-
	IGF2-AS: E2				FL2	6 (67%)	3 (33%)	*	0	7 (100%)	-	-
2	chr11:2,146,313	rs1003483	T	G	FL1	4 (50%)	4 (50%)	*	0	3 (100%)	-	-
	IGF2-AS: E2				FL2	4 (57%)	3 (43%)	*	0	2 (100%)	-	-
3	chr11:2,131,906	rs11042774	T	C	FL1	5 (56%)	4 (44%)	*	86 (4%)	2185 (96%)	-	-
	IGF2(2): E4				FL2	3 (27%)	8 (73%)	*	69 (3%)	2167 (97%)	-	-
4	chr11:2,131,663	rs7129583	G	A	FL1	4 (29%)	10 (71%)	*	33 (1%)	2836 (99%)	-	-
	IGF2(2): E4				FL2	11 (58%)	8 (42%)	*	62 (2%)	2832 (98%)	-	-
5	chr11:2,131,311	rs3168310	C	G	FL1	3 (75%)	1 (25%)	*	3 (0%)	1082 (100%)	-	-
	IGF2(2): E4				FL2	3 (50%)	3 (50%)	*	2 (0%)	1060 (100%)	-	-
6	chr11:2,129,862	rs181296097	C	T	FL1	3 (33%)	6 (67%)	*	9 (0%)	3190 (100%)	-	-
	IGF2(2): E4				FL2	3 (33%)	6 (67%)	*	11 (0%)	3132 (100%)	-	-
7	chr11:2,148,544	rs3741208	A	G	Li4	8 (53%)	7 (47%)	*	-	-	0	1 (100%)
	IGF2-AS: E3				Li5	12 (46%)	14 (54%)	*	-	-	0	2 (100%)
					Li6	16 (52%)	15 (48%)	*	-	-	-	-
					Li7	11 (46%)	13 (54%)	*	-	-	-	-
8	chr11:2,147,784	rs10770125	A	G	Li2	7 (70%)	3 (30%)	*	-	-	264 (56%)	205 (44%)
	IGF2(6): E3				Li4	4 (44%)	5 (56%)	*	-	-	65 (44%)	84 (56%)
					Li5	9 (60%)	6 (40%)	*	-	-	208 (57%)	159 (43%)
					Li6	6 (43%)	8 (57%)	*	-	-	125 (43%)	163 (57%)
					Li7	6 (38%)	10 (63%)	*	-	-	232 (50%)	233 (50%)
					Li8	2 (50%)	2 (50%)	*	-	-	111 (54%)	93 (46%)
					Li9	5 (50%)	4 (40%)	*	-	-	196 (54%)	165 (46%)
9	chr11:2,132,404	rs680	T	C	Li2	11 (52%)	10 (48%)	*	-	-	433 (42%)	604 (58%)
	IGF2(6): E6				Li6	11 (52%)	10 (48%)	*	-	-	247 (40%)	366 (60%)
					Li7	13 (65%)	7 (35%)	*	-	-	364 (57%)	276 (43%)
					Li9	7 (54%)	6 (46%)	*	-	-	340 (64%)	194 (36%)
5	chr11:2,131,311	rs3168310	C	G	Li2	6 (35%)	11 (65%)	*	-	-	535 (36%)	935 (64%)
	IGF2(6): E6				Li4	4 (50%)	4 (50%)	*	-	-	229 (44%)	294 (56%)
					Li6	7 (39%)	11 (61%)	*	-	-	334 (39%)	531 (61%)
					Li7	10 (53%)	9 (47%)	*	-	-	616 (56%)	484 (44%)
					Li9	2 (33%)	4 (67%)	*	-	-	424 (60%)	281 (40%)
10	chr11:2,129,214	rs2585	T	C	Li2	9 (45%)	10 (50%)	*	-	-	761 (42%)	1043 (58%)
	IGF2(6): E6				Li4	12 (52%)	11 (48%)	*	-	-	335 (44%)	429 (56%)
					Li7	13 (50%)	13 (50%)	*	-	-	657 (59%)	456 (41%)
					Li9	8 (44%)	10 (56%)	*	-	-	584 (55%)	475 (45%)

He, heterozygous SNP

Supplementary Table 6. Read counts of informative SNPs within the *IGF2* transcripts of the human muscle and lung

SNP	Genomic coordinate & Transcript: Exon	rs ID	Allele		Tissue	gDNA		He	S.muscle (mRNA)		Lung (mRNA)	
			Ref	Alt		Ref	Alt		Ref	Alt	Ref	Alt
1	chr11:2,146,388	rs1003484	A	G	SM1	26 (58%)	19 (42%)	*	1 (100%)	0	-	-
	IGF2-AS: E2				SM3	11 (46%)	13 (54%)	*	0	0	-	-
					SM5	24 (52%)	22 (48%)	*	0	0	-	-
11	chr11:2,132,928 IGF2(1,2): E4	rs1006752092	G	A	SM3	2 (29%)	5 (71%)	*	4 (4%)	88 (96%)	-	-
12	chr11:2,130,876	rs57156844	C	T	SM4	7 (39%)	11 (61%)	*	2 (3%)	77 (97%)	-	-
	IGF2(1,2): E4				SM5	14 (67%)	7 (33%)	*	103 (99%)	1 (1%)	-	-
13	chr11:2,130,822 IGF2(1,2): E4	rs3802971	G	A	SM3	23 (70%)	10 (30%)	*	2 (7%)	25 (93%)	-	-
14	chr11:2,130,611 IGF2(1,2): E4	rs117403190	A	C	SM2	25 (64%)	14 (36%)	*	56 (100%)	0	-	-
15	chr11:2,129,467	rs7873	T	C	SM2	22 (45%)	27 (55%)	*	77 (95%)	4 (5%)	-	-
	IGF2(1,2): E4				SM3	18 (62%)	11 (38%)	*	115 (100%)	0	-	-
					SM4	30 (64%)	17 (36%)	*	113 (97%)	3 (3%)	-	-
					SM5	44 (59%)	30 (41%)	*	226 (97%)	4 (2%)	-	-
1	chr11:2,146,388	rs1003484	A	G	Lu2	22 (71%)	9 (29%)	*	-	-	0	0
	IGF2-AS: E2				Lu3	31 (74%)	11 (26%)	*	-	-	0	1 (100%)
16	chr11:2,132,961 IGF2(1,2): E4	rs3741214	G	A	Lu2	24 (44%)	30 (55%)	*	-	-	1 (3%)	31 (97%)
9	chr11:2,132,404	rs680	T	C	Lu2	23 (62%)	14 (38%)	*	-	-	4 (9%)	41 (91%)
	IGF2(1,2): E4				Lu3	30 (55%)	25 (45%)	*	-	-	1 (2%)	62 (98%)
					Lu5	11 (44%)	14 (56%)	*	-	-	34 (92%)	3 (8%)
5	chr11:2,131,311	rs3168310	C	G	Lu2	60 (47%)	67 (53%)	*	-	-	1 (1%)	83 (99%)
	IGF2(1,2): E4				Lu3	43 (49%)	44 (51%)	*	-	-	3 (3%)	83 (97%)
					Lu5	61 (59%)	43 (41%)	*	-	-	38 (95%)	2 (5%)
10	chr11:2,129,214	rs2585	T	C	Lu2	44 (69%)	19 (30%)	*	-	-	8 (4%)	188 (95%)
	IGF2(1,2): E4				Lu3	53 (54%)	46 (46%)	*	-	-	0	80 (100%)
					Lu5	50 (54%)	43 (46%)	*	-	-	97 (99%)	1 (1%)
					Lu6	59 (61%)	37 (38%)	*	-	-	6 (4%)	165 (96%)

He, heterozygous SNP; S.muscle, smooth muscle.

Supplementary Table 7. Sequencing depth of Hi-C data

Total reads processed for Fig. 9 using Hi-C datasets (PRJNA482496: fetal and adult livers, GSE143288: adult skeletal muscle)

Bamaxiang pig fetal liver	Run	Mates	No. of Trimmed reads
replicate1	SRR7585922	R1	470106697
		R2	470106697
replicate2	SRR7591440	R1	352633186
		R2	352633186
replicate3	SRR7591441	R1	352442625
		R2	352442625
total			2350365016

Bamaxiang pig adult liver	Run	Mates	No. of Trimmed reads
replicate1	SRR7585913	R1	497694700
		R2	497694700
replicate2	SRR7591447	R1	498347127
		R2	498347127
replicate3	SRR7585920	R1	455364812
		R2	455364812
total			2902813278

Large White pig (2-week-old) skeletal muscle	Run	Mates	No. of Trimmed reads
replicate1	SRR10764658	R1	552247539
		R2	552247539
replicate2	SRR10764659	R1	569433835
		R2	569433835
total			2243362748

Total reads processed for Fig. S16 using a Hi-C dataset (GSE166346: fetal muscle)

Luchuan pig fetal muscle	Run	Mates	No. of Trimmed reads
replicate1	SRR13651468	R1	1601432743
		R2	1601432743
total			3202865486

Supplementary Table 8. Datasets used in this study

Profiling gene regulatory elements

Accession	Sequencing	Subject	Figure
GSE158430	ATAC-seq/ ChIP-seq (H3K27ac, H3K4me3, and CTCF)/ RNA-seq	pig (6-month-old) liver and skeletal muscle	Fig. 2
GSE143288	ATAC-seq/ ChIP-seq (H3K27ac and H3K4me3)/ RNA-seq	pig (2-week-old) liver	Fig. 3
GSE153452	CTCF-seq	pig embryonic fibroblasts	S.Fig. 3
GSE155324	CTCF-seq	human lymphoblasts	S.Fig. 3

Analyses of tissue-specific and developmental stage-specific expression

Accession	Sequencing	Subject	Figure
PRJEB44486 †	RNA-seq	pig embryonic liver and skeletal muscle	Fig. 4
PRJEB44486 †	RNA-seq	pig (1-day-old) liver and skeletal muscle	Fig. 4
GSE77776 ‡	RNA-seq	pig (60-day-old) liver and skeletal muscle	Fig. 4
PRJNA493166	RNA-seq	pig (180-day-old) liver and skeletal muscle	Fig. 4
GSE63634 ‡	RNA-seq	human fetal liver	Fig. 6
hum0158.v2 ††, ‡	RNA-seq	human adult liver	Fig. 6
SRP166862 ‡	RNA-seq	human adult smooth muscle	Fig. 6
PRJNA395106 ‡	RNA-seq	human adult lung	Fig. 6
GSE158430	RNA-seq	pig (6-month-old) multiple tissues	S.Fig. 2
Listed in S.Fig. 4	RNA-seq	pig liver in various developmental stages	S.Fig. 4
Listed in S.Fig. 5	RNA-seq	pig skeletal muscle in various developmental stages	S.Fig. 5
GSE120795	RNA-seq	human adult multiple tissues	S.Fig. 6

† Dataset found in https://data.faang.org/dataset. †† Controlled access. Datasets other than † and †† are found in either https://www.ncbi.nlm.nih.gov/gds or https://www.ncbi.nlm.nih.gov/.

‡ Dataset also used in Analyses of allele-specific expression

Analyses of allele-specific expression

Accession	Sequencing	Subject	Figure
PRJNA309108 / GSE77776	WGS / RNA-seq	pig (60-day-old) liver and skeletal muscle	Fig. 5
GSE63634	H3K4me1 ChIP-seq / RNA-seq	human fetal liver	Fig. 7
hum0158.v2	WGS / RNA-seq	human adult liver	Fig. 7
SRP163897 / SRP166862	WES / RNA-seq	human adult smooth muscle	Fig. 8
PRJNA395106	WES / RNA-seq	human adult lung	Fig. 8
PRJNA597972	RNA-seq #	pig (28-day-old) liver	S.Fig. 4

Biallelic tendency was analyzed using an RNA-seq dataset.

Hi-C data processing

Accession	Sequencing	Subject	Figure
PRJNA482496	Hi-C	fetal and adult pig livers	Fig. 9
GSE143288	Hi-C	2-week-old pig skeletal muscle	Fig. 9
GSE166346	Hi-C	fetal pig muscle	S.Fig. 18
